# Prevalence and distribution of depression in Ghana: A nationwide survey using Patient Health Questionnaire-9 (PHQ-9)

**DOI:** 10.1371/journal.pmen.0000498

**Published:** 2026-06-25

**Authors:** Leveana Gyimah, Edmond Banafo Nartey, Emmanuel Parbie Abbeyquaye, Abraham Hodgson, Pascal Kingsley Mwin, Philip Teg-Nefaah Tabong, Benjamin Demah Nuertey, Joana Ansong, Amma Mpomaa Boadu, Patricia Rarau, Stefan Savin, Cheick Diallo, Kouamivi Agboyigbor

**Affiliations:** 1 Disease Prevention Cluster, WHO Ghana Country Office, Accra, Ghana; 2 Department of Child Health, 37 Military Hospital, Accra, Ghana; 3 Research Division, Ghana Health Service, Accra, Ghana; 4 School of Public Health, University of Ghana, Legon, Accra, Ghana; 5 Department of Mental Health, Institutional Care Division, Ghana Health Service, Accra, Ghana; 6 Department of Noncommunicable Diseases and Mental Health, WHO Headquarters, Geneva, Switzerland; 7 Department of Noncommunicable Diseases, WHO-African Regional Office, Brazzaville, Congo; Uganda Martyrs University, UGANDA

## Abstract

Depression often runs a chronic course and substantially impairs an individual’s occupational potential and quality of life. The incident cases of depression worldwide increased from 17.2 million in 1990 to 25.8 million in 2017, an increase of 49.86%. Studies among the general population have revealed that socio-demographic factors such as older age, marginalization, and female gender, were important risk. This study sought to provide the prevalence and distribution of cases of depression in Ghana using a nationwide cross-sectional methodology. A total of 5202 participants aged 18–69 years were sampled from all regions of Ghana using a multi-stage cluster sampling approach. Probable depression was assessed using the Patient Health Questionnaire-9 (PHQ-9) as part of the WHO STEPWise survey for non-communicable disease (NCD) risk factors. Data was collected face-to-face and electronically using a pre-loaded structured questionnaire translated into Ga, Ewe, Dagbanli and Twi for inclusivity. Among the respondents, 49.5% were females (n = 2,580), and the majority (44%) were aged 18–29 years. Urban residents constituted 59.9%, while rural dwellers made up 40.1%. Overall, 67.8% of respondents reported no depression, while 32.2% had probable depression. Female respondents were 35% more likely to experience probable depression compared to men (aOR=1.35, 95% CI: 1.12–1.64, p = 0.002). Higher education levels were associated with a 48% lower likelihood of probable depression (aOR=0.52, 95% CI: 0.31–0.86, p = 0.011). Wealthier individuals were 42% more likely to experience probable depression than the poorest respondents (aOR=1.42, 95% CI: 1.05–1.93, p = 0.001). Respondents from households with two or three members were 26% and 29% less likely to report probable depression, respectively, compared to households with more members. Probable depression affects nearly one-third of Ghanaians, with women and individuals from wealthier households disproportionately impacted. Higher education and smaller household sizes are protective factors. These findings underscore the need for mental health interventions targeting socio-demographic disparities.

## Introduction

Depression is a common mental health condition in the general population [1] characterised by sadness, loss of interest or pleasure, feelings of guilt or low self-worth, disturbed sleep or appetite, feelings of tiredness, and poor concentration [2]. In its most severe form, depression can lead to death by suicide [3] and an increased risk of mortality. Its pathophysiology is multifactorial, involving dysregulation of monoamine neurotransmission, hypothalamic–pituitary–adrenal axis activity, neuroinflammatory processes, and altered neuroplasticity, shaped by genetic, psychological, and social determinants [4].Clinically, depression includes several diagnostic entities, notably major depressive disorder, postpartum depression, and depressive syndromes occurring with post‑traumatic stress disorder and other conditions [4]. Depression often runs a chronic course and substantially impairs an individual’s occupational potential and quality of life [5]. The World Health Organisation (WHO) has reported that depression is ranked second in global disease burden and is one of the priority conditions covered by the WHO’s Mental Health Gap Action Programme [6].

The global prevalence of depression and depressive symptoms has been increasing in recent decades. The number of incident cases of depression worldwide increased from 172 million in 1990 to 25.8 million in 2017, representing an increase of 49.86% [7]. The lifetime prevalence of depression ranges from 20% to 25% in women compared to 7% to 12% in men [8]. Population‑based studies across West Africa report substantial burdens of depressive symptoms in countries with sociodemographic profiles comparable to Ghana, including Nigeria, Côte d’Ivoire, Burkina Faso, and Togo [9,10] Shared contextual determinants, such as poverty, informal employment, urbanisation, and limited mental health service coverage, shape population risk and detection. However, prevalence estimates vary widely because of differences in sampling frames, instruments, and thresholds [7].

When comparing sexes, years of healthy life lost due to disability (YLD) rates in females were nearly twice as high as in males for depressive disorders [11]. Prior studies among the general population have revealed that socio-demographic factors such as older age, parents’ occupational status, marginalization [2], female gender [3], lower education levels of parents, and living conditions of parents [12] were important risk factors for depression. In addition, psychosocial risk factors for depression are family disputes, low socioeconomic status, and undesirable academic performance [13]. A recent study has identified marginalization and socio-economic status as risk factors for depression [14]. Depression has also been reported to have a positive correlation with quality of life among the general population [15]. There is a wide variation in depression levels among the Ghanaian population. Though data on the prevalence of depression among different population groups in Ghana is available, there is no reliable data on national prevalence in the general population.

A community-based cross-sectional study in one region estimated the prevalence of depression to be 25.2% and was noted to be associated with other mental health challenges and comorbidities [16]. Another study of university students determined a prevalence of 57% for mild to extremely severe depression [17]. Among females with fertility challenges, depression was found to be as high as 62%, with a positive correlation with increasing age and duration of infertility [18]. Living environments may play a role in the degree or presence of depression; however, no significant difference was observed in a 12-month study in Ghana and South Africa among urban and rural dwellers [19].

Few studies provide nationally representative data, limiting regional comparability. Using the Patient Health Questionnaire‑9, a brief and widely validated screening tool employed across African settings [20–22], enables standardized measurement and facilitates comparison of Ghana’s burden with neighbouring countries to inform policy‑relevant planning decisions.

With this wide variation in prevalence depending on the population under consideration, nationally representative data were therefore required to help direct policy implementation strategies by the health authorities. The study aimed to determine the prevalence of probable depression in Ghana to provide a baseline estimate that will inform the design and implementation of targeted interventions aimed at reducing the burden of probable depression in the country.

## Materials and methods

### Study area

The study was conducted in the 16 regions of Ghana. The regions were stratified into three ecological zones, namely Coastal (Western, Central, Greater Accra and Volta), Middle (Eastern, Western North, Ashanti, Bono, Bono East, Oti, and Ahafo) and Northern (Northern, Savannah, North East, Upper East, and Upper West).

### Study design

This study was a nationwide cross-sectional survey that employed a quantitative research methodology to systematically collect and analyse data on depressive symptoms among adults. The design enabled the estimation of prevalence rates and the examination of associations between socio-demographic factors and probable depression across diverse geographic regions and population subgroups in Ghana. By using standardized instruments and structured data collection procedures, the study sought to generate generalizable, empirically robust findings that can inform public health policy and intervention strategies.

### Study population

The population for the study was adults aged 18–69 years residing in Ghana.

The inclusion criteria for participants were randomly selected adults (male and female) aged 18–69 years residing in Ghana, who were permanent residents of selected households. The definition for an eligible household member was a person who has been in the household for at least 6 months in the study area were included in the survey.

The following individuals were excluded from the survey:

- Those who were 18–69 years but were severely ill (as determined by a health professional, this referred to individuals with acute medical conditions or functional impairments that prevented meaningful participation in the interview, including those who had severe cognitive impairment, or were experiencing acute psychiatric or medical emergencies) and unable to respond to an interview at the time of data collection.
- Persons in institutionalized settings, such as in hotels, nursing homes, and other institutions
- Members of the household who had been away for more than six months or those who were in the household for less than six months and were not going to stay longer.

### Sample size determination

The sample size for this study was determined using the Cochran formula [23]. The prevalence of depression was assumed to be 50% since this is the first nationwide survey to be conducted in Ghana. The calculated sample size was adjusted for 80% response rate to get a sample size of 5775. At the end of the survey, 5202 participants were included in the analysis (90% response rate).

### Sampling technique

A multi-stage cluster sampling design was employed for this study. The primary sampling units (PSUs) or clusters were randomly selected proportional to population size, followed by the random selection of households and, subsequently, of individual survey participants. Each region of Ghana was first stratified into urban and rural areas based on population distribution. Within each stratum, 15 enumeration areas (EAs) were randomly selected. In each selected EA, households were randomly sampled using the 2020 Population and Housing Census frame provided by the Ghana Statistical Service. A household listing was conducted, after which one eligible participant was randomly selected from each household using the eSTEPS sampling software.

Eligibility criteria required that individuals be permanent residents of the household for at least six months before the survey. Individuals who declined to provide informed consent or who were residing in institutional settings, such as hospitals, were excluded from the study.

### Data collection tool

The study adopted the structured questionnaire for the WHO STEPWise survey for NCD risk factors. The prevalence of probable depressive disorder was assessed using the Patient Health Questionnaire 9 (PHQ-9), which has been shown to be valid and reliable for assessing a current depressive disorder in the general population [24] and in Ghana [13]. The PHQ-9 is a self-reported questionnaire that is composed of nine items that correspond to the DSM-IV diagnostic criteria for a major depressive episode, including thoughts of death and suicide.

The internal consistency of the PHQ-9 in the present study was assessed using Cronbach’s alpha (α = 0.83) and McDonald’s omega (ω = 0.80). Both coefficients exceed the conventional threshold of 0.70 for research instruments, indicating satisfactory internal reliability within the study sample. Furthermore, a confirmatory factor analysis (CFA) was conducted to examine the unidimensionality of the PHQ-9. The one-factor model demonstrated a good fit to the data (χ²/df = 1.87, Comparative Fit Index [CFI] = 0.96, Tucker–Lewis Index [TLI] = 0.95, Root Mean Square Error of Approximation [RMSEA] = 0.045; 90% CI: 0.030–0.060). These fit indices collectively support a unidimensional factor structure of the PHQ-9 in this study population.

### Data collection procedure

Face-to-face data collection was conducted using a pre-loaded structured questionnaire on an android tablet for on-site electronic data collection. The questionnaire was translated into four languages: Ga, Ewe, Dagbanli and Twi. The questions were asked based on the participants’ language preference. The data was collected between 18^th^ July 2023 and 17^th^ August 2023 in all 16 regions of the country. Data collectors were trained on the conduct of the assessment by experienced mental health professionals. Their skills were reinforced through role-plays to identify gaps and provide support to avoid errors or inconsistencies in the data collection process. The questions on the PHQ-9 were read out to the participants, who were to indicate the duration they had been experiencing each symptom if it applied to them over the past 2 weeks.

### Data analysis

STATA version 16 was used to analyse the data. Descriptive statistical methods were first used to summarise the distribution of the data across demographic characteristics of the study participants, and the prevalence of probable depression. In determining the prevalence of probable depression, a PHQ-9 score was used. The PHQ-9 response set was standardized by asking the number of days in the past 2 weeks the respondent had experienced a particular depressive symptom. The modified response set was converted back to the original response set: 0–1 day = “not at all,” 2–6 days = “several days,” 7–11 days = “more than half the days,” and 12–14 days = “nearly every day,” with points (0–3) assigned to each category, respectively. The presence and severity of symptoms on a 4-point Likert scale over the past two weeks, adhering to the Diagnostic and Statistical Manual criteria [4]. This scoring ranges from 0 to 27 for the PHQ-9, with higher scores indicating greater symptom severity. After data weighting, the sampling design was catered by preceding every command in STATA with the survey command (svy).

For this analysis, we combined the no probable depression and minimal probable depression and classified them as no depression. We then combined mild (5–9), moderate (10–14), moderately severe (15–19), and severe (20–27) as probable depression. S1 Table shows the individual questions in the PHQ-9 and their prevalence in that population,

The PHQ-9 has been extensively validated as a screening and diagnostic tool for depressive disorders across diverse populations. Globally, a cut-off score of 10 is widely accepted as the optimal threshold for identifying probable cases of major depressive disorder (MDD). At this cut-off, the instrument demonstrates high diagnostic accuracy, with a pooled sensitivity of approximately 88% and specificity of approximately 88% in distinguishing individuals with MDD from those without [25,26]. These metrics indicate that the PHQ-9 has strong discriminatory capacity and minimal misclassification error at this threshold.

Several studies have also examined the performance of the PHQ-9 in African and Ghanaian contexts. In a Ghanaian postpartum population, Weobong and colleagues found that a cut-off score of 10 provided the best balance between sensitivity and specificity when validated against the Comprehensive Psychopathological Rating Scale [27]. The area under the ROC curve (AUC) was 0.90 (95% CI: 0.81–0.98), indicating excellent diagnostic performance. At this cut-off, the sensitivity was 94%, and the specificity was 75%, confirming that the PHQ-9 effectively discriminated between cases and non-cases of depression within this population.

Further, studies in sub-Saharan Africa have consistently supported the PHQ-9’s reliability as a diagnostic and severity measure. For example, Cholera and colleagues reported sensitivities ranging between 84% and 94% and specificities between 75% and 88% [28], depending on the population and language version administered.

Collectively, these findings demonstrate that the PHQ-9 maintains robust sensitivity and specificity values across cultural and linguistic contexts, including Ghana, supporting its use as a valid and reliable instrument for assessing depressive symptoms and classifying depression severity in community and clinical samples.

The wealth index was constructed based on data on household assets and possessions. Prior to its computation, Principal Component Analysis (PCA) was performed to identify household assets and possessions that effectively discriminated between wealthier and poorer households. The first principal component, which accounted for the largest proportion of variance in asset ownership, was used to generate household wealth scores. These scores were subsequently categorized into quintiles, ranging from the poorest to the richest, to represent the relative socioeconomic status of participants. Descriptive statistics were computed to summarize the data, with frequencies and percentages used to describe participants’ socio-demographic characteristics and the prevalence of probable depression. Subsequently, binary logistic regression analysis was performed to assess the association between participants’ socio-demographic factors and probable depression status. Multicollinearity diagnostics were conducted using the Variance Inflation Factor (VIF). The mean VIF value was 1.72, with all individual VIFs below 2.5, which is well within the commonly accepted threshold of < 5. These results indicate that no significant multicollinearity existed among the independent variables included in the regression model.

### Ethical approval and consent to participate

The study was approved by the Ghana Health Service Ethics Review Committee (GHS-ERC 032/08/22). Written informed consent was obtained from all participants.

## Results

### Description of demographic factors and depression among the respondents

The total number of respondents for this study was 5202 with 34% of them being females. The age ranged from 18 to 69 years. The overall prevalence rate of probable depression was 32.2% in the population. There were no significant differences in the prevalence of probable depression among the various age groups (p = 0.08). Female respondents had a higher prevalence of probable depression (n = 2580, 34.0%; p = 0.010) than men. Respondents with higher education had less prevalence of probable depression than those with lower educational level (n = 424, 18.5%; p < 0.001). Religion showed significant differences (p = 0.042), with higher probable depression prevalence among traditionalists/spiritualists (n = 183, 37.2%) and Muslims (n = 1310, 35.6%) than Christians (n = 3606, 29.4%) and those with no indicated religion (n = 103, 25.0%). Those currently married had a lower proportion of probable depression (n = 2442, 28.6%, p = 0.017) than those who are separated/divorced/widowed (37.6%, n = 488) and cohabiting (n = 320, 36.9%). Assessing depression using the wealth index showed significant differences with a p-0.001 among the rich (n = 942, 37.4%), middle (n = 1063, 34.5%), and richest (n = 778, 32.9%) compared to the poor (n = 1163, 29.7%) and poorest (n = 1256, 23.9%), respectively. There were no significant differences in the prevalence of probable depression among the various ethnic groups and areas of residence, i.e., urban versus rural. However, there were regional variations in the prevalence of probable depression, with the highest in Northern (n = 335, 47.8%) and Western North (n = 147, 42.9%), while the lowest was in the Upper East (n = 220, 16.3%) and Upper West (n = 151, 17.9%) Regions. Table 1 shows the demographic factors and depression rate among the participants.

**Table 1:** This is a summary visualisation of the demographic factors and depression among the respondents.

Variable	Weighted frequency	Probable depression		p-value
		n	% (95% CI)	
**Age group**				**0.806**
18-29	2291	698	30.4 (26.7-34.5)	
30-44	1637	512	31.3 (28.5-34.2)	
45-59	929	303	32.6 (29.1-36.3)	
60-69	345	108	31.4 (26.7-36.5)	
**Sex**				**0.010**
Men	2622	745	28.4 (25.0-32.0)	
Female	2580	876	34.0 (31.5-36.5)	
**Education**				**<0.001**
No education	893	333	37.2 (32.1-42.7)	
Primary	1095	375	34.2 (30.7-38.0)	
Junior high school	1597	470	29.5 (26.4-32.7)	
Senior high school	1193	365	30.6 (25.7-35.9)	
Higher	424	78	18.5 (14.4-23.5)	
**Ethnicity**				**0.056**
Akan	2048	620	30.3 (27.5-33.2)	
Ga/dangme	300	68	22.5 (16.9-29.4)	
Ewe	689	240	34.8 (29.9-40.0)	
Guan	208	49	23.8 (16.8-32.4)	
Mole-dagbani	1039	374	36 (30.5-41.9)	
Grusi	170	34	20.1 (14.0-27.9)	
Gurma	232	82	35.3 (27.9-43.4)	
Mande	55	15	27 (10.8-53.0)	
Other	461	139	30.2 (20.5-42.0)	
**Religion**				**0.042**
Christian	3606	1060	29.4 (27.4-31.4)	
Muslim	1310	467	35.6 (29.8-41.9)	
Traditionalist/spiritual	183	68	37.2 (29.6-45.6)	
None	103	26	25 (15.8-37.1)	
**Marital status**				**0.017**
Never married	1952	622	31.8 (27.5-36.6)	
Currently married	2442	698	28.6 (26.4-30.8)	
Separate/ divorced/ widowed	488	183	37.6 (33.0-42.5)	
Cohabitating	320	118	36.9 (30.7-43.6)	
**Occupation**				**0.070**
Unemployed	1433	479	33.4 (28.2-39.1)	
Government employee	232	51	21.8 (16.1-28.8)	
Non-government employee	516	171	33.1 (27.1-39.8)	
Self employed	2971	912	30.7 (28.6-32.9)	
Retired	50	8	16 (9.1-26.6)	
**Household size**				**0.138**
1 member	938	315	33.6 (30.7-36.7)	
2 members	1824	519	28.4 (26.1-30.9)	
3 members	1718	515	30 (26.8-33.3)	
4 + Members	722	272	37.7 (27.2-49.5)	
**Wealth index**				**<0.001**
Poorest	1256	298	23.9 (20.3-27.9)	
Poor	1163	345	29.7 (26.0-33.7)	
Middle	1063	365	34.5 (29.8-39.4)	
Rich	942	354	37.4 (33.3-41.7)	
Richest	778	259	32.9 (28.5-37.7)	
**Residence**				**0.907**
Rural	2088	647	31 (28.3-33.8)	
Urban	3114	974	31.3 (28.0-34.8)	
**Region**				**<0.001**
Western	260	67	26.0 (20.5-32.4)	
Central	532	165	31.0 (25.8-36.8)	
Greater Accra	853	183	21.4 (16.6-27.3)	
Volta	262	105	40.2 (33.0-47.9)	
Eastern	480	191	39.8 (35.0-44.9)	
Ashanti	801	201	25.1 (21.4-29.2)	
Western north	147	63	42.9 (36.3-49.8)	
Ahafo	82	22	26.5 (17.8-37.3)	
Bono	196	57	29.3 (21.7-38.2)	
Bono east	190	67	35.2 (26.1-45.4)	
Oti	117	48	40.8 (34.2-47.7)	
Northern	701	335	47.8 (38.6-57.1)	
Savannah	112	26	23.2 (18.2-29.1)	
North east	98	27	27.4 (19.9-36.4)	
Upper east	220	36	16.3 (10.8-23.8)	
Upper west	151	27	17.9 (12.9-24.3)	

### Probable depression status among the respondents

**Fig 1:**
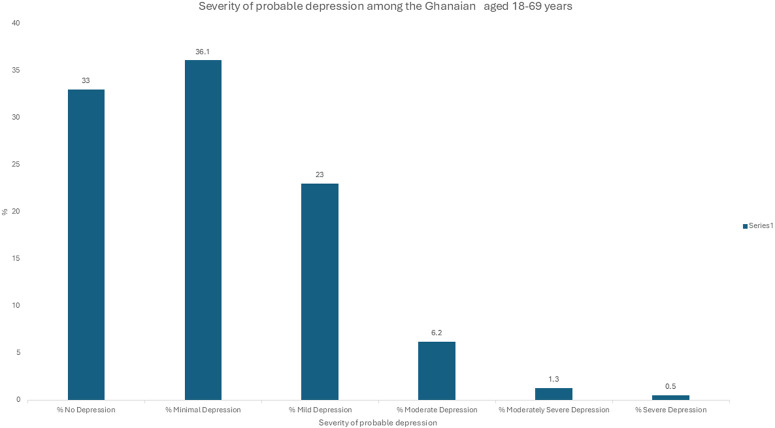
The severity of probable depression among respondents who were screened using the PHQ-9 questionnaire.

**Fig 2:**
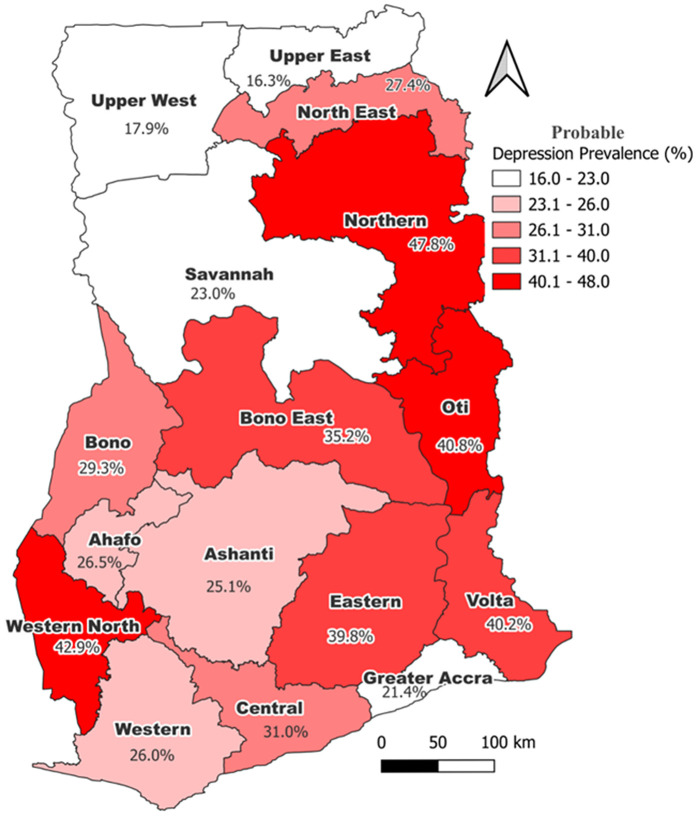
This is the regional distribution of the prevalence of probable depression. Source: Created with ArcGIS (base map shapefile: Ghana Statistical Service: https://data.humdata.org/dataset/cod-ab-gha).

The study found 1674 (32.2%, 95% CI: 30.9-33.5) of the respondents were depressed, while 3528 (67.8%, 95% CI: 66.5-69.1) were not. Fig 1 shows the severity of the probable depression per percentage of respondents and those who screened negative while Fig 2 shows the regional distribution of the prevalence probable depression.

### Associated factors and the odds of probable depression among the respondents

Female respondents were 35% more likely to experience probable depression compared to men (aOR=1.35, 95% CI: 1.12–1.64, p = 0.002). Respondents with higher education were 48% less likely to experience probable depression compared to those with no education (aOR=0.52, 95% CI: 0.31–0.86, p = 0.011). Respondents with household size two and three 26% and 29% [(aOR=0.74, 95% CI: 0.62-0.89), p = 0.001); (aOR=0.71, (95% CI: 0.55-0.90), p = 0.005)] were less likely to experience probable depression, respectively. Respondents that were rich were 42% more likely to experience probable depression compared to those in the poorest category (aOR=1.42, 95% CI: 1.05–1.93). Table 2 shows the associated factors and the odds of probable depression among the participants.

**Table 2:** The associated factors and the odds of probable depression among the respondents.

Variable	cOR (95% CI), p-value	aOR (95% CI), p-value
**Age group**		
18-29	**Ref.**	
30-44	1.04 (0.85-1.27), 0.709	
45-59	1.11 (0.87-1.41), 0.409	
60-69	1.04 (0.78-1.4), 0.767	
**Sex**		
Men	**Ref.**	**Ref.**
Women	1.3 (1.06-1.58), 0.010	1.35 (1.12-1.64), 0.002
**Education**		
No education	**Ref.**	**Ref.**
Primary	0.88 (0.67-1.15), 0.340	0.93 (0.69-1.26), 0.63
Junior high school	0.7 (0.56-0.89), 0.003	0.81 (0.6-1.09), 0.158
Senior high school	0.74 (0.53-1.03), 0.075	0.92 (0.62-1.36), 0.671
Higher	0.38 (0.26-0.56), < 0.001	0.52 (0.31-0.86), 0.011
**Ethnicity**		
Akan	**Ref.**	
Ga/dangme	0.67 (0.46-0.98), 0.040	
Ewe	1.23 (0.95-1.59), 0.115	
Guan	0.72 (0.46-1.13), 0.153	
Mole-dagbani	1.3 (0.98-1.72), 0.070	
Grusi	0.58 (0.37-0.91), 0.018	
Gurma	1.26 (0.87-1.81), 0.221	
Mande	0.85 (0.28-2.63), 0.781	
Other	1 (0.58-1.71), 0.987	
**Religion**		
Christian	1.25 (0.71-2.22), 0.442	
Muslim	1.66 (0.89-3.1), 0.108	
Traditionalist/spiritual	1.78 (0.93-3.4), 0.080	
None	**Ref.**	
**Marital status**		
Never married	**Ref.**	
Currently married	0.86 (0.68-1.07), 0.177	
Separate/ divorced/ widowed	1.29 (0.97-1.72), 0.084	
Cohabitating	1.25 (0.89-1.76), 0.201	
**Occupation**		
Unemployed	**Ref.**	**Ref.**
Government employee	**0.55 (0.35-0.89), 0.015**	0.89 (0.53-1.49), 0.658
Non-government employee	0.99 (0.67-1.46), 0.948	1.32 (0.88-1.99), 0.175
Self employed	0.88 (0.69-1.13), 0.317	0.92 (0.72-1.17), 0.503
Retired	**0.38 (0.19-0.76), 0.007**	0.61 (0.31-1.19), 0.148
**Household size**		
1 member	**Ref.**	**Ref.**
2 members	**0.78 (0.66-0.93), 0.006**	**0.74 (0.62-0.89), 0.001**
3 members	0.84 (0.69-1.03), 0.094	**0.71 (0.55-0.90), 0.005**
4 + members	1.19 (0.72-1.98), 0.491	0.93 (0.59-1.47), 0.759
**Wealth index**		
Poorest	**Ref.**	**Ref.**
Poor	**1.35 (1.03-1.76), 0.029**	1.15 (0.87-1.52), 0.318
Middle	**1.68 (1.26-2.24), < 0.001**	1.32 (0.98-1.79), 0.066
Rich	**1.9 (1.44-2.51), < 0.001**	1.42 (1.05-1.93), 0.022
Richest	**1.56 (1.17-2.09), 0.003**	1.2 (0.85-1.70), 0.291
**Residence**		
Rural	**Ref.**	
Urban	1.01 (0.82-1.25), 0.907	
**Region**		
Western	**Ref.**	**Ref.**
Central	1.28 (0.85-1.92), 0.232	1.35 (0.89-2.06), 0.159
Greater Accra	0.78 (0.5-1.21), 0.266	0.83 (0.52-1.32), 0.437
Volta	**1.91 (1.23-2.98), 0.004**	**2.00 (1.26-3.18), 0.003**
Eastern	**1.88 (1.3-2.74), 0.001**	**2.00 (1.36-2.96), < 0.001**
Ashanti	0.96 (0.66-1.39), 0.812	1.00 (0.68-1.49), 0.983
Western north	**2.14 (1.41-3.25), < 0.001**	**2.25 (1.44-3.51), < 0.001**
Ahafo	1.02 (0.57-1.85), 0.939	0.99 (0.55-1.78), 0.976
Bono	1.18 (0.71-1.96), 0.523	1.19 (0.70-2.02), 0.524
Bono east	1.54 (0.91-2.62), 0.108	1.55 (0.91-2.63), 0.106
Oti	**1.96 (1.29-2.98), 0.002**	**2.00 (1.28-3.14), 0.003**
Northern	**2.61 (1.6-4.25), < 0.001**	**2.59 (1.61-4.18), < 0.001**
Savannah	0.86 (0.55-1.33), 0.495	0.85 (0.52-1.40), 0.522
North east	1.07 (0.64-1.8), 0.796	1.02 (0.58-1.81), 0.944
Upper east	**0.55 (0.32-0.98), 0.041**	**0.55 (0.30-10), 0.048**
Upper west	0.62 (0.38-1.02), 0.060	0.64 (0.36-1.12), 0.118

## Discussion

This study identified significant patterns and associated factors of probable depression among respondents, providing critical insights into the burden of probable depression in the population. It was estimated that 32.2% of respondents experienced probable depression, while 67.8% were not. Women were disproportionately affected, with a prevalence rate of 34% compared to men, and women were found to be 35% more likely to experience probable depression. These findings agree with data from Liu and colleagues who revealed that women do bear a heavy burden of depression and anxiety [29], reporting that more women are diagnosed with depression than men, though our observed rates were higher than global averages.

The association of gender with depression prevalence could also be linked to the unique societal pressures women face, including caregiving roles and economic inequalities that exist. Meanwhile, men may not be aware of their status or underreport due to their health-seeking behaviour, stigma around mental health, and societal expectations of men. Interestingly, our study findings of men having lower rates of prevalence are in contrast with studies among university students, which showed that female students were more psychologically stable than their male counterparts and therefore had lower levels of probable depression and other mental health challenges [17]. This may be due to men in the general population who are facing real-life challenges, whilst female students in the university are more likely to also receive more social support than their male counterparts.

Again, age was not significantly associated with probable depression, as the prevalence remained relatively consistent across age groups. The findings of the study are in contrast with other studies [16,18,29], which reported a positive correlation between age and depression. The lack of variation by age in this study suggests that depression may be driven by broader societal factors rather than age-related stressors; there is a need for interventions that target all age groups equally.

Socioeconomic factors also played a significant role in depression. Respondents with higher levels of education were 48% less likely to experience probable depression compared to those with no education. This aligns with research conducted among adults with tertiary education where 68% were less likely to experience depression [16] and therefore highlights education as a protective factor due to its association with improved coping mechanisms and access to resources. Conversely, respondents from wealthier households were 42% more likely to experience probable depression compared to those in the poorest category. This finding stands in sharp contrast to existing research, which has consistently shown that individuals with higher wealth are less likely to experience probable depression than those with lower economic status [16,30,31]. The observation of a higher prevalence of depression among wealthier individuals and urban residents is somewhat counterintuitive, as lower socioeconomic status is typically associated with increased risk of depression in many settings. However, several plausible explanations and alternative hypotheses may account for this pattern in the Ghanaian context.

First, occupational stress may contribute to elevated depressive symptoms among wealthier and urban individuals. Urban residents and higher-income earners often occupy more demanding or high-responsibility jobs, which can involve longer working hours, high performance expectations, and job insecurity—all of which are established risk factors for depression.

Second, social comparison and lifestyle pressures may play a role. Individuals in wealthier or urban settings may be more exposed to social and material comparisons through media, social networks, or community interactions. Such comparisons can generate feelings of inadequacy, stress, or dissatisfaction, potentially increasing the risk of depressive symptoms despite greater material wealth.

Third, reporting bias should be considered. Individuals with higher education or urban residency may have greater mental health literacy and awareness of depressive symptoms, leading to more accurate or candid reporting on self-administered tools like the PHQ-9. Conversely, underreporting may occur among rural or lower-income participants due to stigma, lower health literacy, or cultural differences in symptom expression.

Fourth, urban environments may be associated with reduced social cohesion, crowding, and environmental stressors, which could independently contribute to higher depression prevalence. This aligns with prior studies in sub-Saharan Africa that have observed similar urban–rural gradients in mental health outcomes.

Finally, this may also be due to analytical nuance, as the wealth index reflects relative household assets, not income or financial security, and the indices may capture urbanicity and consumption patterns, which could partly explain the association.

These alternative explanations underscore the need for context-specific investigations into the social, occupational, and cultural determinants of depression in Ghana. Future research, particularly longitudinal or qualitative studies, could help disentangle these mechanisms and inform targeted interventions for both urban and rural populations.

Household size is a complex social determinant of mental health that may influence depression risk through both protective and adverse pathways [32–34]

Household size was another factor associated with probable depression. Respondents with household sizes of two or three were 26% and 29% less likely to experience probable depression, respectively. Smaller household sizes may foster stronger interpersonal connections and provide a more stable support system, which can mitigate the risk of depression. In our study, household size showed a graded association with depression. Compared with one‑person households, living in households of two or three members was associated with significantly lower odds of depression after adjustment (AOR = 0.74–0.71), suggesting a protective effect of shared living, possibly through social support and reduced isolation. In contrast, households with four or more members showed no significant association with depression, indicating that potential benefits of social support may be offset by economic strain or caregiving burden. These findings highlight the non‑linear and context‑specific role of household size in probable depression risk in Ghana.[35]. Household size is a contextual social determinant of depression rather than a direct causal factor.

Marital status and religion were also significant predictors of depression. Married respondents had the lowest prevalence of depression, while separated, divorced, or widowed individuals reported the highest prevalence (37.6%). These findings also align with existing research that shows that individuals who are not married, separated, divorced, or widowed have double the odds of getting depressed [30]. This finding underscores the protective effect of marriage and social support on mental health, consistent with existing literature. Traditionalists and spiritualists experience the highest prevalence of depression among religious groups (37.2%), suggesting that cultural beliefs and practices may influence mental health outcomes and help-seeking behaviour.

The Northern, Oti, and Western North regions were found to have the highest estimated prevalence of depression in this study. These regional estimates may reflect contextual stressors, healthcare access, or post-conflict/ or economic vulnerabilities.

The overall prevalence of probable depression in this study (32.2%) highlights a significant public health concern. The findings underscore the urgent need for gender-sensitive, culturally appropriate mental health interventions and policies. Efforts should focus on reducing stigma, improving mental health literacy, and addressing barriers to accessing care. Additionally, targeted interventions for vulnerable groups, such as women, individuals with low education levels, and the economically disadvantaged, are essential for reducing the burden of probable depression in Ghana. These findings also have implications for other low- and middle-income countries (LMICs), where similar socioeconomic and cultural factors may influence mental health outcomes.

## Conclusions

Probable depression is common among the Ghanaian population, especially in women. No significant differences were observed in the prevalence of probable depression among different age groups in this study population. This study highlights a significant prevalence of probable depression (32.2%) among the population in Ghana, with notable disparities across gender, socioeconomic, educational, marital, and religious groups. Women, individuals with lower education levels, and separated, divorced, or widowed individuals were disproportionately affected, while smaller household sizes and higher education levels appeared to be protective factors.

The findings underscore the urgent need for comprehensive, gender-sensitive, and culturally appropriate mental health interventions. Addressing barriers to access, stigma reduction, and the integration of mental health care into primary health systems are critical to reducing the burden of depression. Additionally, targeted support for vulnerable groups and the strengthening of social and economic safety nets are essential for creating a mentally healthier society.

### Implications of the findings

The findings of this study have significant public health, clinical, and policy implications for Ghana. Establishing the prevalence of probable depression among adults aged 18–69 years provides essential baseline data that can guide the design, implementation, and evaluation of targeted mental health interventions at both national and subnational levels. By quantifying the magnitude and distribution of depressive symptoms across socio-demographic groups, this study offers empirical evidence necessary for resource allocation, capacity building, and service planning within Ghana’s mental health system.

From a public health perspective, the results highlight the need to integrate mental health screening and care into primary health care services, particularly through the Community-based Health Planning and Services (CHPS) initiative. Early detection and management of probable depression at the community level can help reduce the long-term social and economic burden associated with untreated mental disorders.

At the policy level, the study underscores the importance of strengthening the implementation of Ghana’s Mental Health Policy and scaling up evidence-based programs for mental health promotion and stigma reduction. The identification of socio-demographic factors associated with depression further provides actionable insights for targeted interventions—such as psychosocial support for vulnerable populations, workplace mental health programs, and gender-sensitive approaches to care.

Finally, the study establishes a robust epidemiological foundation for longitudinal monitoring of probable depression trends. Future studies can use this baseline to assess the impact of interventions over time and to track progress toward achieving national and global mental health targets, including the Sustainable Development Goals (SDG 3.4) on reducing the burden of mental illness.

### Recommendations

There is an urgent need to increase awareness among health care providers and the public of depression and other mental health conditions in the general population. Screening of the general population for (probable) depression on contact with the health system will be essential through community structures in providing a comprehensive service. Strengthen referral pathways for persons who may have signs and symptoms suggestive of depressive disorders through the screening process and provision of appropriate interventions by the mental health practitioner for individuals who are linked to care.

Care for depression needs to be strengthened nationwide through capacity building and delivery of psychological therapy such as Cognitive Behavioural Therapy (CBT) through primary care and community settings, alongside evidence‑based pharmacotherapy, including selective serotonin reuptake inhibitors (SSRI), serotonin–norepinephrine reuptake inhibitors (SNRI), tricyclic antidepressants(TCA), and monoamine oxidase inhibitors (MAO-I) guided by severity, comorbidity, availability, and patient preference, with referral pathways for complex cases nationally.

It is imperative to expand access to affordable mental health services, particularly for vulnerable populations such as those with low education levels, separated or widowed individuals, and economically disadvantaged groups. Building the capacity of religious, traditional, and community leaders as gatekeepers to recognise common signs of depression and linkage to care is essential in reducing the burden of the condition and its potential complication of suicide and suicidal behaviour. This will promote collaboration with orthodox facilities in line with the Mental Health Act, 846 (2012) which emphasizes community-based care and support for mental health care as opposed to institutional care.

Social support programs should be developed to specifically target individuals who are separated, divorced, or widowed, as these life events are associated with increased vulnerability to (probable) depression and other adverse mental health outcomes. Such programs could include peer support groups, counselling services, and community engagement activities to provide emotional support, reduce social isolation, and enhance coping mechanisms.

In addition, empowerment initiatives are recommended to address the financial and economic stressors that can contribute to psychological distress. These may include financial literacy training, vocational skills development, and employment facilitation programs, which can improve individuals’ economic independence and reduce stress related to financial insecurity. By integrating social support and economic empowerment strategies, interventions can more effectively mitigate the multifactorial risk factors for (probable) depression and promote overall mental well-being among vulnerable populations.

### Strengths and limitations

This study has several limitations that should be considered when interpreting the findings.First, the data were collected through self-reported measures, which may be subject to recall and social desirability biases. Participants may have underreported or overreported depressive symptoms or health-related behaviours due to memory lapses or the desire to provide socially acceptable responses. Although trained interviewers and standardized data collection procedures were employed to minimize these biases, their potential influence on the findings cannot be entirely excluded.

Second, the cross-sectional design of the study limits the ability to infer causal relationships between socio-demographic factors and depression. The observed associations should therefore be interpreted as correlational rather than indicative of temporal or causal effects. Longitudinal studies would be required to establish causality and to track changes in depressive symptoms over time.

Third, the study relied on the PHQ-9, a self-report screening tool, without additional objective clinical validation through diagnostic interviews or corroboration with medical records. Although the PHQ-9 has demonstrated strong psychometric properties and has been validated in Ghanaian populations, the absence of a structured clinical assessment may introduce misclassification bias—potentially overestimating or underestimating the true prevalence of probable depression.

Additionally, although the survey was conducted nationwide and included participants from all regions of Ghana, the sample was not fully representative of the underlying national population across all sociodemographic characteristics. As a result, the prevalence estimates reported should be interpreted with caution and should not be considered fully generalizable to the entire Ghanaian population. Nonetheless, the broad geographic coverage and large sample size provide valuable insights into the distribution of depressive symptoms and regional patterns of burden.

Lastly, formal multi-group measurement invariance testing across sex, education, or region was not conducted and should be considered in future analyses.

Despite these limitations, the study provides important baseline evidence on the prevalence and determinants of probable depression in Ghana. It offers valuable insights to inform future intervention design and longitudinal research.

## Supporting information

S1 Table Analysis of Probable Depression among survey respondents for the nationwide STEPS survey in Ghana. (DOCX)

S1 Text Patient Health Questionnaire (PHQ-9). (PDF)

S2 Text WHO STEPS Instrument. (DOCX)

S1 Checklist Inclusivity in global research questionnaire. (DOCX)
