# Supplementary material for: Prevalence and distribution of depression in Ghana: A nationwide survey using Patient Health Questionnaire-9 (PHQ-9)
Source: PLOS Ment Health. 2026 Jun 25;3(6):e0000498. doi: 10.1371/journal.pmen.0000498 (PMC13298969; doi:10.1371/journal.pmen.0000498)
Supplement: S1 Table — (DOCX) [file pmen.0000498.s001.docx]

**Analysis of Probable Depression among survey respondents for the nationwide STEPS survey in Ghana**

**PHQ1**

| **Percentage with little interest or pleasure in doing things** | | | | | | | | | |
| --- | --- | --- | --- | --- | --- | --- | --- | --- | --- |
| Age Group  (years) | **Men** | | | | | | | | |
|  | n |  | | | |  | | | |
|  |  | % Not at all | 95% CI | % Several days | 95% CI | % More than half the days | 95% CI | % Nearly everyday | 95% CI |
| 18-29 | 550 | 65.1 | 59.5-70.7 | 22.6 | 17.9-27.2 | 10.2 | 6.5-14.0 | 2.1 | 0.8-3.5 |
| 30-44 | 721 | 69.8 | 66.0-73.6 | 18.6 | 15.4-21.7 | 10.5 | 7.6-13.3 | 1.2 | 0.0-2.5 |
| 45-59 | 519 | 72.0 | 66.6-77.4 | 17.7 | 12.6-22.8 | 7.4 | 4.6-10.1 | 2.9 | 1.4-4.4 |
| 60-69 | 232 | 78.1 | 71.8-84.3 | 10.4 | 6.1-14.6 | 7.1 | 3.7-10.6 | 4.5 | 0.1-8.8 |
| **18-69** | **2022** | **68.5** | **65.5-71.5** | **19.8** | **17.4-22.2** | **9.6** | **7.6-11.6** | **2.1** | **1.3-2.9** |

| **Percentage with little interest or pleasure in doing things** | | | | | | | | | |
| --- | --- | --- | --- | --- | --- | --- | --- | --- | --- |
| Age Group  (years) | **Women** | | | | | | | | |
|  | n |  | | | |  | | | |
|  |  | % Not at all | 95% CI | % Several days | 95% CI | % More than half the days | 95% CI | % Nearly everyday | 95% CI |
| 18-29 | 873 | 67.1 | 63.2-70.9 | 21.8 | 18.1-25.4 | 9.2 | 6.8-11.5 | 2.0 | 0.9-3.1 |
| 30-44 | 1393 | 65.5 | 62.1-69.0 | 21.9 | 19.1-24.7 | 9.7 | 7.7-11.6 | 2.8 | 1.7-4.0 |
| 45-59 | 812 | 67.6 | 63.7-71.5 | 21.5 | 18.0-25.1 | 8.0 | 5.7-10.3 | 2.9 | 1.4-4.3 |
| 60-69 | 338 | 72.0 | 65.7-78.2 | 17.3 | 12.6-22.1 | 7.9 | 3.7-12.1 | 2.8 | 0.7-4.9 |
| **18-69** | **3416** | **67.0** | **64.8-69.2** | **21.5** | **19.5-23.5** | **9.0** | **7.7-10.3** | **2.5** | **1.8-3.1** |

| **Percentage with little interest or pleasure in doing things** | | | | | | | | | |
| --- | --- | --- | --- | --- | --- | --- | --- | --- | --- |
| Age Group  (years) | **Both Sexes** | | | | | | | | |
|  | n |  | | | |  | | | |
|  |  | % Not at all | 95% CI | % Several days | 95% CI | % More than half the days | 95% CI | % Nearly everyday | 95% CI |
| 18-29 | 1423 | 66.0 | 62.6-69.5 | 22.2 | 19.1-25.3 | 9.7 | 7.4-12.1 | 2.1 | 1.2-3.0 |
| 30-44 | 2114 | 67.6 | 65.1-70.0 | 20.3 | 18.2-22.4 | 10.1 | 8.4-11.7 | 2.1 | 1.2-2.9 |
| 45-59 | 1331 | 69.8 | 66.5-73.1 | 19.7 | 16.6-22.7 | 7.7 | 5.9-9.4 | 2.9 | 1.9-3.9 |
| 60-69 | 570 | 75.0 | 70.5-79.6 | 13.8 | 10.6-17.1 | 7.5 | 4.6-10.4 | 3.6 | 1.2-6.1 |
| **18-69** | **5438** | **67.7** | **65.8-69.7** | **20.6** | **19.0-22.2** | **9.3** | **8.1-10.5** | **2.3** | **1.8-2.8** |

**PHQ2**

| **Percentage feeling down, depressed or hopeless** | | | | | | | | | |
| --- | --- | --- | --- | --- | --- | --- | --- | --- | --- |
| Age Group  (years) | **Men** | | | | | | | | |
|  | n |  | | | |  | | | |
|  |  | % Not at all | 95% CI | % Several days | 95% CI | % More than half the days | 95% CI | % Nearly everyday | 95% CI |
| 18-29 | 550 | 67.1 | 61.4-72.8 | 17.4 | 13.3-21.5 | 13.3 | 8.9-17.7 | 2.3 | 1.0-3.5 |
| 30-44 | 721 | 67.4 | 63.5-71.2 | 21.8 | 18.3-25.3 | 8.6 | 6.2-11.0 | 2.3 | 0.6-3.9 |
| 45-59 | 519 | 73.7 | 69.1-78.2 | 16.6 | 12.9-20.2 | 7.4 | 4.6-10.2 | 2.4 | 1.0-3.8 |
| 60-69 | 232 | 68.3 | 60.2-76.3 | 15.4 | 10.4-20.3 | 11.4 | 6.1-16.7 | 5.0 | 0.4-9.5 |
| **18-69** | **2022** | **68.4** | **65.4-71.3** | **18.4** | **16.1-20.7** | **10.8** | **8.5-13.0** | **2.5** | **1.6-3.3** |

| **Percentage feeling down, depressed or hopeless** | | | | | | | | | |
| --- | --- | --- | --- | --- | --- | --- | --- | --- | --- |
| Age Group  (years) | **Women** | | | | | | | | |
|  | n |  | | | |  | | | |
|  |  | % Not at all | 95% CI | % Several days | 95% CI | % More than half the days | 95% CI | % Nearly everyday | 95% CI |
| 18-29 | 873 | 64.7 | 60.5-69.0 | 23.2 | 19.6-26.8 | 8.6 | 6.2-11.1 | 3.4 | 1.8-5.0 |
| 30-44 | 1393 | 61.7 | 58.4-65.0 | 25.7 | 22.8-28.7 | 9.5 | 7.6-11.4 | 3.1 | 2.0-4.1 |
| 45-59 | 812 | 63.3 | 58.9-67.8 | 21.8 | 18.2-25.4 | 11.0 | 7.9-14.0 | 3.9 | 2.3-5.5 |
| 60-69 | 338 | 64.9 | 57.8-72.0 | 21.1 | 15.4-26.8 | 9.7 | 5.3-14.1 | 4.3 | 1.4-7.3 |
| **18-69** | **3416** | **63.5** | **61.1-65.9** | **23.6** | **21.6-25.7** | **9.4** | **8.0-10.9** | **3.5** | **2.6-4.3** |

| **Percentage feeling down, depressed or hopeless** | | | | | | | | | |
| --- | --- | --- | --- | --- | --- | --- | --- | --- | --- |
| Age Group  (years) | **Both Sexes** | | | | | | | | |
|  | n |  | | | |  | | | |
|  |  | % Not at all | 95% CI | % Several days | 95% CI | % More than half the days | 95% CI | % Nearly everyday | 95% CI |
| 18-29 | 1423 | 65.9 | 62.3-69.6 | 20.2 | 17.5-22.9 | 11.1 | 8.4-13.8 | 2.8 | 1.8-3.8 |
| 30-44 | 2114 | 64.4 | 62.0-66.9 | 23.8 | 21.6-26.0 | 9.1 | 7.6-10.6 | 2.7 | 1.8-3.6 |
| 45-59 | 1331 | 68.4 | 64.8-72.0 | 19.2 | 16.5-22.0 | 9.2 | 7.1-11.4 | 3.2 | 2.1-4.2 |
| 60-69 | 570 | 66.6 | 61.6-71.6 | 18.2 | 14.4-22.0 | 10.6 | 7.3-13.8 | 4.7 | 1.9-7.4 |
| **18-69** | **5438** | **66.0** | **64.0-67.9** | **21.0** | **19.4-22.6** | **10.1** | **8.7-11.5** | **2.9** | **2.3-3.6** |

**PHQ3**

| **Percentage with trouble falling or staying asleep, or sleeping too much** | | | | | | | | | |
| --- | --- | --- | --- | --- | --- | --- | --- | --- | --- |
| Age Group  (years) | **Men** | | | | | | | | |
|  | n |  | | | |  | | | |
|  |  | % Not at all | 95% CI | % Several days | 95% CI | % More than half the days | 95% CI | % Nearly everyday | 95% CI |
| 18-29 | 550 | 77.1 | 69.9-84.2 | 13.4 | 8.3-18.5 | 7.0 | 4.2-9.9 | 2.5 | 0.6-4.3 |
| 30-44 | 721 | 72.4 | 68.2-76.6 | 13.9 | 10.9-16.8 | 10.0 | 7.3-12.7 | 3.8 | 2.2-5.4 |
| 45-59 | 519 | 69.0 | 64.1-73.9 | 18.0 | 14.1-21.9 | 10.1 | 6.8-13.3 | 3.0 | 1.3-4.6 |
| 60-69 | 232 | 68.2 | 61.6-74.8 | 16.8 | 11.5-22.1 | 8.6 | 4.6-12.6 | 6.4 | 1.3-11.5 |
| **18-69** | **2022** | **73.7** | **70.4-77.1** | **14.5** | **12.0-17.1** | **8.5** | **7.0-10.1** | **3.2** | **2.2-4.2** |

| **Percentage with trouble falling or staying asleep, or sleeping too much** | | | | | | | | | |
| --- | --- | --- | --- | --- | --- | --- | --- | --- | --- |
| Age Group  (years) | **Women** | | | | | | | | |
|  | n |  | | | |  | | | |
|  |  | % Not at all | 95% CI | % Several days | 95% CI | % More than half the days | 95% CI | % Nearly everyday | 95% CI |
| 18-29 | 873 | 71.3 | 67.2-75.4 | 19.8 | 16.2-23.4 | 5.9 | 3.9-7.8 | 3.0 | 1.6-4.4 |
| 30-44 | 1393 | 66.2 | 63.1-69.3 | 18.4 | 15.6-21.2 | 11.7 | 9.5-13.9 | 3.7 | 2.7-4.8 |
| 45-59 | 812 | 62.4 | 58.2-66.5 | 21.7 | 18.3-25.2 | 10.2 | 7.6-12.9 | 5.7 | 3.5-7.8 |
| 60-69 | 338 | 58.2 | 51.2-65.2 | 20.2 | 14.8-25.6 | 13.2 | 8.4-18.0 | 8.4 | 4.7-12.1 |
| **18-69** | **3416** | **67.2** | **65.0-69.4** | **19.7** | **17.8-21.6** | **9.0** | **7.8-10.2** | **4.1** | **3.2-4.9** |

| **Percentage with trouble falling or staying asleep, or sleeping too much** | | | | | | | | | |
| --- | --- | --- | --- | --- | --- | --- | --- | --- | --- |
| Age Group  (years) | **Both Sexes** | | | | | | | | |
|  | n |  | | | |  | | | |
|  |  | % Not at all | 95% CI | % Several days | 95% CI | % More than half the days | 95% CI | % Nearly everyday | 95% CI |
| 18-29 | 1423 | 74.3 | 70.4-78.3 | 16.4 | 13.5-19.4 | 6.5 | 4.8-8.2 | 2.7 | 1.5-3.9 |
| 30-44 | 2114 | 69.2 | 66.6-71.8 | 16.2 | 14.1-18.3 | 10.9 | 9.1-12.6 | 3.7 | 2.8-4.7 |
| 45-59 | 1331 | 65.6 | 62.3-68.9 | 19.9 | 17.1-22.7 | 10.1 | 7.9-12.4 | 4.3 | 3.0-5.7 |
| 60-69 | 570 | 63.2 | 58.6-67.8 | 18.5 | 14.7-22.3 | 10.9 | 7.7-14.1 | 7.4 | 3.9-10.9 |
| **18-69** | **5438** | **70.5** | **68.4-72.6** | **17.1** | **15.5-18.8** | **8.8** | **7.8-9.7** | **3.6** | **2.9-4.3** |

**PHQ4**

| **Percentage experience feeling tired or having little energy** | | | | | | | | | |
| --- | --- | --- | --- | --- | --- | --- | --- | --- | --- |
| Age Group  (years) | **Men** | | | | | | | | |
|  | n |  | | | |  | | | |
|  |  | % Not at all | 95% CI | % Several days | 95% CI | % More than half the days | 95% CI | % Nearly everyday | 95% CI |
| 18-29 | 550 | 62.9 | 56.5-69.3 | 23.9 | 18.3-29.4 | 10.9 | 7.7-14.0 | 2.4 | 1.0-3.8 |
| 30-44 | 721 | 62.7 | 58.6-66.9 | 22.9 | 19.1-26.7 | 11.2 | 8.4-13.9 | 3.2 | 1.6-4.8 |
| 45-59 | 519 | 61.8 | 56.6-66.9 | 24.9 | 20.4-29.5 | 8.3 | 5.4-11.2 | 5.0 | 2.8-7.3 |
| 60-69 | 232 | 64.0 | 56.1-71.9 | 21.3 | 13.7-28.9 | 11.8 | 7.2-16.4 | 2.9 | 0.8-5.0 |
| **18-69** | **2022** | **62.7** | **59.6-65.9** | **23.6** | **20.9-26.3** | **10.6** | **8.8-12.4** | **3.1** | **2.2-4.0** |

| **Percentage experience feeling tired or having little energy** | | | | | | | | | |
| --- | --- | --- | --- | --- | --- | --- | --- | --- | --- |
| Age Group  (years) | **Women** | | | | | | | | |
|  | n |  | | | |  | | | |
|  |  | % Not at all | 95% CI | % Several days | 95% CI | % More than half the days | 95% CI | % Nearly everyday | 95% CI |
| 18-29 | 873 | 58.4 | 54.0-62.9 | 25.7 | 21.9-29.5 | 11.8 | 9.0-14.7 | 4.0 | 2.4-5.7 |
| 30-44 | 1393 | 53.0 | 49.2-56.7 | 28.5 | 25.1-32.0 | 14.4 | 10.4-18.4 | 4.1 | 2.9-5.3 |
| 45-59 | 812 | 54.4 | 49.9-58.9 | 28.7 | 24.6-32.9 | 11.1 | 8.6-13.5 | 5.8 | 3.5-8.1 |
| 60-69 | 338 | 56.6 | 49.8-63.4 | 22.3 | 17.3-27.3 | 13.0 | 8.7-17.4 | 8.1 | 4.2-11.9 |
| **18-69** | **3416** | **55.8** | **53.4-58.2** | **26.9** | **24.8-29.1** | **12.6** | **10.7-14.5** | **4.6** | **3.7-5.6** |

| **Percentage experience feeling tired or having little energy** | | | | | | | | | |
| --- | --- | --- | --- | --- | --- | --- | --- | --- | --- |
| Age Group  (years) | **Both Sexes** | | | | | | | | |
|  | n |  | | | |  | | | |
|  |  | % Not at all | 95% CI | % Several days | 95% CI | % More than half the days | 95% CI | % Nearly everyday | 95% CI |
| 18-29 | 1423 | 60.8 | 56.8-64.7 | 24.7 | 21.2-28.2 | 11.3 | 9.0-13.6 | 3.2 | 2.1-4.2 |
| 30-44 | 2114 | 57.7 | 55.1-60.3 | 25.8 | 23.2-28.4 | 12.8 | 10.5-15.2 | 3.7 | 2.6-4.7 |
| 45-59 | 1331 | 58.0 | 54.6-61.3 | 26.9 | 23.7-30.0 | 9.7 | 7.9-11.6 | 5.4 | 3.9-7.0 |
| 60-69 | 570 | 60.3 | 55.1-65.5 | 21.8 | 16.9-26.7 | 12.4 | 9.3-15.5 | 5.5 | 3.3-7.7 |
| **18-69** | **5438** | **59.3** | **57.2-61.4** | **25.3** | **23.6-26.9** | **11.6** | **10.2-12.9** | **3.9** | **3.2-4.6** |

**PHQ5**

| **Percentage experience poor appetite or overeating** | | | | | | | | | |
| --- | --- | --- | --- | --- | --- | --- | --- | --- | --- |
| Age Group  (years) | **Men** | | | | | | | | |
|  | n |  | | | |  | | | |
|  |  | % Not at all | 95% CI | % Several days | 95% CI | % More than half the days | 95% CI | % Nearly everyday | 95% CI |
| 18-29 | 550 | 75.9 | 69.9-81.9 | 15.4 | 11.1-19.8 | 7.6 | 3.6-11.6 | 1.1 | 0.1-2.0 |
| 30-44 | 721 | 78.8 | 75.2-82.3 | 14.2 | 11.1-17.3 | 6.4 | 4.2-8.5 | 0.7 | 0.1-1.2 |
| 45-59 | 519 | 78.4 | 73.8-82.9 | 14.6 | 11.0-18.3 | 5.1 | 2.7-7.4 | 1.9 | 0.3-3.5 |
| 60-69 | 232 | 87.2 | 82.6-91.8 | 9.2 | 5.2-13.1 | 2.6 | 0.7-4.5 | 1.1 | 0.0-2.3 |
| **18-69** | **2022** | **77.9** | **74.6-81.2** | **14.6** | **12.2-16.9** | **6.5** | **4.4-8.6** | **1.1** | **0.5-1.6** |

| **Percentage experience poor appetite or overeating** | | | | | | | | | |
| --- | --- | --- | --- | --- | --- | --- | --- | --- | --- |
| Age Group  (years) | **Women** | | | | | | | | |
|  | n |  | | | |  | | | |
|  |  | % Not at all | 95% CI | % Several days | 95% CI | % More than half the days | 95% CI | % Nearly everyday | 95% CI |
| 18-29 | 873 | 68.2 | 63.8-72.6 | 20.6 | 16.8-24.5 | 8.5 | 6.1-11.0 | 2.7 | 1.3-4.1 |
| 30-44 | 1393 | 68.6 | 65.3-71.9 | 18.7 | 16.2-21.3 | 10.2 | 7.8-12.6 | 2.4 | 1.4-3.4 |
| 45-59 | 812 | 71.2 | 67.4-75.1 | 16.0 | 13.0-19.0 | 8.4 | 6.0-10.9 | 4.4 | 2.3-6.4 |
| 60-69 | 338 | 74.5 | 68.6-80.3 | 15.1 | 10.7-19.6 | 8.6 | 4.4-12.7 | 1.8 | 0.1-3.6 |
| **18-69** | **3416** | **69.3** | **66.8-71.7** | **18.8** | **16.9-20.8** | **9.1** | **7.6-10.5** | **2.9** | **2.0-3.7** |

| **Percentage experience poor appetite or overeating** | | | | | | | | | |
| --- | --- | --- | --- | --- | --- | --- | --- | --- | --- |
| Age Group  (years) | **Both Sexes** | | | | | | | | |
|  | n |  | | | |  | | | |
|  |  | % Not at all | 95% CI | % Several days | 95% CI | % More than half the days | 95% CI | % Nearly everyday | 95% CI |
| 18-29 | 1423 | 72.2 | 68.7-75.7 | 17.9 | 15.1-20.7 | 8.0 | 5.7-10.4 | 1.8 | 1.0-2.7 |
| 30-44 | 2114 | 73.5 | 71.1-76.0 | 16.6 | 14.5-18.6 | 8.4 | 6.6-10.1 | 1.6 | 1.0-2.1 |
| 45-59 | 1331 | 74.7 | 71.7-77.8 | 15.3 | 12.9-17.7 | 6.8 | 5.1-8.5 | 3.2 | 1.9-4.5 |
| 60-69 | 570 | 80.9 | 76.7-85.0 | 12.1 | 8.9-15.4 | 5.6 | 3.3-7.9 | 1.4 | 0.4-2.5 |
| **18-69** | **5438** | **73.6** | **71.6-75.6** | **16.7** | **15.2-18.1** | **7.8** | **6.5-9.0** | **2.0** | **1.5-2.5** |

**PHQ6**

| **Feel bad about yourself or that you are a failure or have let yourself or your family down** | | | | | | | | | |
| --- | --- | --- | --- | --- | --- | --- | --- | --- | --- |
| Age Group  (years) | **Men** | | | | | | | | |
|  | n |  | | | |  | | | |
|  |  | % Not at all | 95% CI | % Several days | 95% CI | % More than half the days | 95% CI | % Nearly everyday | 95% CI |
| 18-29 | 550 | 76.1 | 71.0-81.2 | 15.0 | 10.8-19.1 | 6.6 | 3.5-9.6 | 2.4 | 0.3-4.5 |
| 30-44 | 721 | 75.7 | 72.1-79.2 | 14.6 | 11.7-17.6 | 7.2 | 5.0-9.4 | 2.5 | 1.0-4.1 |
| 45-59 | 519 | 75.7 | 71.2-80.2 | 14.6 | 10.8-18.5 | 6.5 | 3.9-9.2 | 3.1 | 1.0-5.2 |
| 60-69 | 232 | 80.0 | 74.0-86.0 | 12.9 | 7.6-18.2 | 5.2 | 2.4-7.9 | 1.9 | 0.0-3.9 |
| **18-69** | **2022** | **76.1** | **73.4-78.8** | **14.7** | **12.4-16.9** | **6.7** | **5.1-8.2** | **2.5** | **1.4-3.7** |

| **Feel bad about yourself or that you are a failure or have let yourself or your family down** | | | | | | | | | |
| --- | --- | --- | --- | --- | --- | --- | --- | --- | --- |
| Age Group  (years) | **Women** | | | | | | | | |
|  | n |  | | | |  | | | |
|  |  | % Not at all | 95% CI | % Several days | 95% CI | % More than half the days | 95% CI | % Nearly everyday | 95% CI |
| 18-29 | 873 | 76.1 | 72.4-79.7 | 14.2 | 11.7-16.7 | 7.1 | 4.7-9.5 | 2.6 | 1.4-3.9 |
| 30-44 | 1393 | 69.6 | 65.7-73.6 | 19.6 | 16.6-22.6 | 8.3 | 5.8-10.8 | 2.5 | 1.6-3.5 |
| 45-59 | 812 | 76.0 | 72.4-79.5 | 15.7 | 12.4-19.0 | 5.7 | 3.8-7.5 | 2.6 | 1.4-3.8 |
| 60-69 | 338 | 81.1 | 76.0-86.2 | 11.4 | 7.3-15.4 | 5.6 | 2.7-8.6 | 1.9 | 0.3-3.6 |
| **18-69** | **3416** | **74.3** | **72.1-76.5** | **16.0** | **14.5-17.6** | **7.1** | **5.7-8.5** | **2.6** | **1.9-3.2** |

| **Feel bad about yourself or that you are a failure or have let yourself or your family down** | | | | | | | | | |
| --- | --- | --- | --- | --- | --- | --- | --- | --- | --- |
| Age Group  (years) | **Both Sexes** | | | | | | | | |
|  | n |  | | | |  | | | |
|  |  | % Not at all | 95% CI | % Several days | 95% CI | % More than half the days | 95% CI | % Nearly everyday | 95% CI |
| 18-29 | 1423 | 76.1 | 73.0-79.1 | 14.6 | 12.2-17.0 | 6.8 | 4.8-8.8 | 2.5 | 1.2-3.8 |
| 30-44 | 2114 | 72.5 | 69.8-75.3 | 17.2 | 15.1-19.3 | 7.7 | 5.8-9.6 | 2.5 | 1.7-3.4 |
| 45-59 | 1331 | 75.8 | 72.9-78.8 | 15.2 | 12.6-17.8 | 6.1 | 4.5-7.7 | 2.9 | 1.7-4.1 |
| 60-69 | 570 | 80.6 | 76.5-84.6 | 12.2 | 8.7-15.6 | 5.4 | 3.4-7.4 | 1.9 | 0.6-3.2 |
| **18-69** | **5438** | **75.2** | **73.3-77.1** | **15.4** | **13.9-16.8** | **6.9** | **5.8-8.0** | **2.5** | **1.9-3.2** |

**PHQ7**

| **Percentage having trouble concentrating on things, such as reading the newspaper or watching television** | | | | | | | | | |
| --- | --- | --- | --- | --- | --- | --- | --- | --- | --- |
| Age Group  (years) | **Men** | | | | | | | | |
|  | n |  | | | |  | | | |
|  |  | % Not at all | 95% CI | % Several days | 95% CI | % More than half the days | 95% CI | % Nearly everyday | 95% CI |
| 18-29 | 550 | 76.7 | 71.3-82.1 | 15.8 | 11.2-20.3 | 5.3 | 2.3-8.3 | 2.2 | 0.2-4.2 |
| 30-44 | 721 | 81.0 | 77.4-84.6 | 10.6 | 7.6-13.6 | 7.2 | 4.8-9.6 | 1.2 | 0.2-2.1 |
| 45-59 | 519 | 82.1 | 78.4-85.9 | 10.7 | 7.7-13.6 | 6.2 | 3.4-8.9 | 1.0 | 0.2-1.8 |
| 60-69 | 232 | 83.1 | 77.3-88.8 | 10.3 | 5.0-15.5 | 6.0 | 2.9-9.2 | 0.6 | 0.0-1.6 |
| **18-69** | **2022** | **79.3** | **76.6-82.1** | **13.0** | **10.6-15.4** | **6.1** | **4.4-7.8** | **1.6** | **0.6-2.6** |

| **Percentage having trouble concentrating on things, such as reading the newspaper or watching television** | | | | | | | | | |
| --- | --- | --- | --- | --- | --- | --- | --- | --- | --- |
| Age Group  (years) | **Women** | | | | | | | | |
|  | n |  | | | |  | | | |
|  |  | % Not at all | 95% CI | % Several days | 95% CI | % More than half the days | 95% CI | % Nearly everyday | 95% CI |
| 18-29 | 873 | 75.9 | 71.9-80.0 | 16.8 | 13.5-20.0 | 5.8 | 3.8-7.8 | 1.5 | 0.5-2.6 |
| 30-44 | 1393 | 74.9 | 71.2-78.6 | 15.2 | 12.6-17.7 | 8.6 | 6.1-11.2 | 1.3 | 0.6-2.0 |
| 45-59 | 812 | 80.3 | 77.0-83.5 | 12.4 | 9.6-15.1 | 5.1 | 3.2-7.0 | 2.3 | 0.9-3.6 |
| 60-69 | 338 | 83.3 | 78.2-88.3 | 8.0 | 4.9-11.2 | 6.4 | 3.1-9.8 | 2.3 | 0.5-4.1 |
| **18-69** | **3416** | **76.8** | **74.6-79.1** | **14.9** | **13.2-16.6** | **6.6** | **5.3-7.9** | **1.6** | **1.1-2.2** |

| **Percentage having trouble concentrating on things, such as reading the newspaper or watching television** | | | | | | | | | |
| --- | --- | --- | --- | --- | --- | --- | --- | --- | --- |
| Age Group  (years) | **Both Sexes** | | | | | | | | |
|  | n |  | | | |  | | | |
|  |  | % Not at all | 95% CI | % Several days | 95% CI | % More than half the days | 95% CI | % Nearly everyday | 95% CI |
| 18-29 | 1423 | 76.3 | 72.9-79.7 | 16.2 | 13.5-19.0 | 5.6 | 3.6-7.6 | 1.9 | 0.7-3.0 |
| 30-44 | 2114 | 77.9 | 75.5-80.2 | 13.0 | 11.1-14.8 | 8.0 | 6.4-9.6 | 1.2 | 0.7-1.8 |
| 45-59 | 1331 | 81.2 | 78.6-83.7 | 11.5 | 9.5-13.5 | 5.6 | 4.0-7.2 | 1.7 | 0.9-2.4 |
| 60-69 | 570 | 83.2 | 79.2-87.1 | 9.2 | 6.1-12.3 | 6.2 | 3.9-8.6 | 1.5 | 0.4-2.5 |
| **18-69** | **5438** | **78.1** | **76.2-80.0** | **13.9** | **12.4-15.5** | **6.4** | **5.3-7.4** | **1.6** | **1.0-2.2** |

**PHQ8**

| **Experience moving or speaking so slowly that other people could have noticed Or the opposite, being so fidgety or restless that you have been moving around a lot more than usual** | | | | | | | | | |
| --- | --- | --- | --- | --- | --- | --- | --- | --- | --- |
| Age Group  (years) | **Men** | | | | | | | | |
|  | n |  | | | |  | | | |
|  |  | % Not at all | 95% CI | % Several days | 95% CI | % More than half the days | 95% CI | % Nearly everyday | 95% CI |
| 18-29 | 550 | 82.2 | 76.7-87.8 | 12.7 | 8.5-16.8 | 3.8 | 1.3-6.2 | 1.3 | 0.0-3.1 |
| 30-44 | 721 | 85.1 | 82.0-88.2 | 9.1 | 6.8-11.4 | 3.7 | 2.0-5.4 | 2.1 | 0.2-4.0 |
| 45-59 | 519 | 85.7 | 82.0-89.3 | 10.0 | 6.5-13.5 | 3.2 | 1.3-5.1 | 1.1 | 0.2-2.0 |
| 60-69 | 232 | 86.4 | 81.5-91.3 | 6.6 | 3.0-10.2 | 5.8 | 2.5-9.2 | 1.2 | 0.0-2.5 |
| **18-69** | **2022** | **84.0** | **80.9-87.0** | **10.8** | **8.6-12.9** | **3.8** | **2.5-5.1** | **1.5** | **0.5-2.5** |

| **Experience moving or speaking so slowly that other people could have noticed Or the opposite, being so fidgety or restless that you have been moving around a lot more than usual** | | | | | | | | | |
| --- | --- | --- | --- | --- | --- | --- | --- | --- | --- |
| Age Group  (years) | **Women** | | | | | | | | |
|  | n |  | | | |  | | | |
|  |  | % Not at all | 95% CI | % Several days | 95% CI | % More than half the days | 95% CI | % Nearly everyday | 95% CI |
| 18-29 | 873 | 81.6 | 78.1-85.1 | 9.9 | 7.5-12.3 | 6.5 | 4.2-8.8 | 2.0 | 0.8-3.1 |
| 30-44 | 1393 | 84.3 | 82.0-86.6 | 9.5 | 7.4-11.5 | 5.4 | 3.5-7.3 | 0.8 | 0.3-1.3 |
| 45-59 | 812 | 83.6 | 80.5-86.8 | 9.0 | 6.7-11.3 | 3.8 | 2.3-5.3 | 3.6 | 1.7-5.4 |
| 60-69 | 338 | 86.4 | 81.5-91.3 | 8.2 | 4.2-12.1 | 4.4 | 1.9-6.8 | 1.1 | 0.0-2.2 |
| **18-69** | **3416** | **83.1** | **81.2-85.1** | **9.5** | **8.1-10.9** | **5.5** | **4.2-6.8** | **1.8** | **1.2-2.5** |

| **Experience moving or speaking so slowly that other people could have noticed Or the opposite, being so fidgety or restless that you have been moving around a lot more than usual** | | | | | | | | | |
| --- | --- | --- | --- | --- | --- | --- | --- | --- | --- |
| Age Group  (years) | **Both Sexes** | | | | | | | | |
|  | n |  | | | |  | | | |
|  |  | % Not at all | 95% CI | % Several days | 95% CI | % More than half the days | 95% CI | % Nearly everyday | 95% CI |
| 18-29 | 1423 | 81.9 | 78.7-85.1 | 11.4 | 8.9-13.8 | 5.1 | 3.5-6.7 | 1.6 | 0.5-2.7 |
| 30-44 | 2114 | 84.7 | 82.7-86.7 | 9.3 | 7.8-10.8 | 4.6 | 3.3-5.9 | 1.4 | 0.5-2.4 |
| 45-59 | 1331 | 84.6 | 82.1-87.1 | 9.5 | 7.5-11.5 | 3.5 | 2.3-4.7 | 2.4 | 1.3-3.4 |
| 60-69 | 570 | 86.4 | 82.8-90.1 | 7.4 | 4.7-10.0 | 5.1 | 2.9-7.3 | 1.1 | 0.3-2.0 |
| **18-69** | **5438** | **83.5** | **81.7-85.4** | **10.1** | **8.9-11.4** | **4.6** | **3.7-5.6** | **1.7** | **1.1-2.3** |

**PHQ9**

| **Have thoughts that you would be better off dead, or of hurting yourself** | | | | | | | | | |
| --- | --- | --- | --- | --- | --- | --- | --- | --- | --- |
| Age Group  (years) | **Men** | | | | | | | | |
|  | n |  | | | |  | | | |
|  |  | % Not at all | 95% CI | % Several days | 95% CI | % More than half the days | 95% CI | % Nearly everyday | 95% CI |
| 18-29 | 550 | 92.2 | 88.7-95.7 | 5.1 | 3.0-7.2 | 2.2 | 0.0-4.5 | 0.4 | 0.0-0.9 |
| 30-44 | 721 | 92.6 | 90.4-94.9 | 4.5 | 2.8-6.2 | 2.4 | 0.9-4.0 | 0.4 | 0.0-0.9 |
| 45-59 | 519 | 94.6 | 92.4-96.8 | 3.9 | 1.9-5.8 | 1.1 | 0.1-2.1 | 0.4 | 0.0-0.8 |
| 60-69 | 232 | 89.6 | 85.1-94.0 | 8.5 | 4.2-12.8 | 1.6 | 0.2-2.9 | 0.3 | 0.0-1.0 |
| **18-69** | **2022** | **92.6** | **90.7-94.4** | **4.9** | **3.8-6.1** | **2.1** | **0.9-3.2** | **0.4** | **0.1-0.7** |

| **Have thoughts that you would be better off dead, or of hurting yourself** | | | | | | | | | |
| --- | --- | --- | --- | --- | --- | --- | --- | --- | --- |
| Age Group  (years) | **Women** | | | | | | | | |
|  | n |  | | | |  | | | |
|  |  | % Not at all | 95% CI | % Several days | 95% CI | % More than half the days | 95% CI | % Nearly everyday | 95% CI |
| 18-29 | 873 | 88.7 | 85.3-92.1 | 6.8 | 4.3-9.3 | 3.6 | 1.9-5.2 | 1.0 | 0.2-1.7 |
| 30-44 | 1393 | 89.5 | 87.4-91.6 | 7.9 | 5.9-9.9 | 1.8 | 1.1-2.5 | 0.8 | 0.3-1.4 |
| 45-59 | 812 | 89.3 | 86.8-91.9 | 8.0 | 5.8-10.2 | 1.6 | 0.7-2.5 | 1.1 | 0.2-1.9 |
| 60-69 | 338 | 91.1 | 87.4-94.9 | 5.0 | 2.4-7.7 | 2.7 | 0.3-5.0 | 1.2 | 0.0-2.7 |
| **18-69** | **3416** | **89.2** | **87.6-90.9** | **7.3** | **6.0-8.6** | **2.6** | **1.8-3.4** | **1.0** | **0.5-1.4** |

| **Have thoughts that you would be better off dead, or of hurting yourself** | | | | | | | | | |
| --- | --- | --- | --- | --- | --- | --- | --- | --- | --- |
| Age Group  (years) | **Both Sexes** | | | | | | | | |
|  | n |  | | | |  | | | |
|  |  | % Not at all | 95% CI | % Several days | 95% CI | % More than half the days | 95% CI | % Nearly everyday | 95% CI |
| 18-29 | 1423 | 90.5 | 88.2-92.9 | 5.9 | 4.3-7.5 | 2.9 | 1.5-4.3 | 0.7 | 0.3-1.1 |
| 30-44 | 2114 | 91.0 | 89.5-92.5 | 6.3 | 5.0-7.6 | 2.1 | 1.2-2.9 | 0.6 | 0.2-1.0 |
| 45-59 | 1331 | 91.9 | 90.2-93.7 | 6.0 | 4.5-7.5 | 1.4 | 0.7-2.0 | 0.7 | 0.2-1.3 |
| 60-69 | 570 | 90.4 | 87.3-93.4 | 6.8 | 4.1-9.5 | 2.1 | 0.8-3.5 | 0.7 | 0.0-1.6 |
| **18-69** | **5438** | **90.9** | **89.8-92.1** | **6.1** | **5.3-6.9** | **2.3** | **1.6-3.0** | **0.7** | **0.4-0.9** |

**Probable Depression**

| **Probable Depression Assessment** | | | | | | | | | | | | | |
| --- | --- | --- | --- | --- | --- | --- | --- | --- | --- | --- | --- | --- | --- |
| Age Group  (years) | **Men** | | | | | | | | | | | | |
|  | n |  | | | |  | | | |  |  |  |  |
|  |  | % No Depression | 95% CI | % Minimal Depression | 95% CI | % Mild Depression | 95% CI | % Moderate Depression | 95% CI | % Moderately Severe Depression | 95% CI | % Severe Depression | 95% CI |
| 18-29 | 550 | 33.1 | 27.1-39.2 | 39.2 | 34.3-44.1 | 20.9 | 14.6-27.1 | 5.4 | 2.9-8.0 | 1.3 | 0.0-3.1 | 0.1 | 0.0-0.3 |
| 30-44 | 721 | 33.5 | 29.2-37.7 | 38.5 | 33.2-43.8 | 21.8 | 18.2-25.3 | 4.6 | 2.9-6.3 | 1.0 | 0.1-2.0 | 0.7 | 0.0-1.9 |
| 45-59 | 519 | 38.1 | 32.7-43.5 | 31.7 | 26.7-36.6 | 25.3 | 20.6-30.1 | 4.1 | 2.1-6.1 | 0.4 | 0.0-1.0 | 0.4 | 0.0-0.8 |
| 60-69 | 232 | 40.3 | 32.8-47.9 | 33.4 | 26.2-40.6 | 18.1 | 12.3-23.8 | 6.4 | 1.8-10.9 | 1.8 | 0.1-3.6 | 0.0 | 0.0-0.0 |
| **18-69** | **2022** | **34.5** | **31.4-37.7** | **37.3** | **34.2-40.4** | **21.7** | **18.4-25.1** | **5.0** | **3.6-6.4** | **1.1** | **0.2-2.0** | **0.3** | **0.0-0.7** |

| **Probable Depression Assessment** | | | | | | | | | | | | | |
| --- | --- | --- | --- | --- | --- | --- | --- | --- | --- | --- | --- | --- | --- |
| Age Group  (years) | **Women** | | | | | | | | | | | | |
|  | n |  | | | |  | | | |  |  |  |  |
|  |  | % No Depression | 95% CI | % Minimal Depression | 95% CI | % Mild Depression | 95% CI | % Moderate Depression | 95% CI | % Moderately Severe Depression | 95% CI | % Severe Depression | 95% CI |
| 18-29 | 873 | 31.0 | 26.7-35.3 | 35.9 | 31.8-40.0 | 24.8 | 21.3-28.4 | 6.5 | 4.4-8.5 | 1.0 | 0.2-1.7 | 0.8 | 0.1-1.5 |
| 30-44 | 1393 | 29.7 | 26.5-32.9 | 35.3 | 32.1-38.6 | 25.1 | 22.3-28.0 | 7.5 | 5.1-10.0 | 1.9 | 1.1-2.7 | 0.4 | 0.0-0.8 |
| 45-59 | 812 | 33.4 | 28.9-37.9 | 33.6 | 29.3-38.0 | 20.6 | 17.1-24.1 | 9.3 | 6.7-11.8 | 2.3 | 0.9-3.7 | 0.8 | 0.1-1.6 |
| 60-69 | 338 | 36.2 | 28.7-43.7 | 28.9 | 22.9-34.8 | 26.2 | 20.1-32.2 | 6.6 | 3.5-9.7 | 1.6 | 0.2-3.0 | 0.6 | 0.0-1.7 |
| **18-69** | **3416** | **31.3** | **29.1-33.6** | **34.9** | **32.6-37.1** | **24.2** | **22.3-26.2** | **7.3** | **6.0-8.7** | **1.6** | **1.1-2.1** | **0.7** | **0.3-1.1** |

| **Probable Depression Assessment** | | | | | | | | | | | | | |
| --- | --- | --- | --- | --- | --- | --- | --- | --- | --- | --- | --- | --- | --- |
| Age Group  (years) | **Both Sexes** | | | | | | | | | | | | |
|  | n |  | | | |  | | | |  |  |  |  |
|  |  | % No Depression | 95% CI | % Minimal Depression | 95% CI | % Mild Depression | 95% CI | % Moderate Depression | 95% CI | % Moderately Severe Depression | 95% CI | % Severe Depression | 95% CI |
| 18-29 | 1423 | 32.1 | 28.5-35.8 | 37.6 | 34.3-40.9 | 22.7 | 19.3-26.2 | 5.9 | 4.1-7.7 | 1.1 | 0.1-2.2 | 0.5 | 0.1-0.8 |
| 30-44 | 2114 | 31.5 | 29.1-33.9 | 36.9 | 33.7-40.0 | 23.5 | 21.2-25.8 | 6.1 | 4.7-7.5 | 1.5 | 0.9-2.1 | 0.6 | 0.0-1.2 |
| 45-59 | 1331 | 35.7 | 31.9-39.5 | 32.7 | 29.1-36.2 | 22.9 | 19.9-26.0 | 6.7 | 5.1-8.4 | 1.4 | 0.6-2.1 | 0.6 | 0.2-1.1 |
| 60-69 | 570 | 38.3 | 33.1-43.4 | 31.2 | 26.8-35.5 | 22.1 | 17.9-26.3 | 6.5 | 3.7-9.3 | 1.7 | 0.6-2.8 | 0.3 | 0.0-0.8 |
| **18-69** | **5438** | **33.0** | **31.0-34.9** | **36.1** | **34.2-38.0** | **23.0** | **21.1-24.8** | **6.2** | **5.2-7.1** | **1.3** | **0.8-1.9** | **0.5** | **0.2-0.8** |
