# Supplementary material for: Prevalence and distribution of depression in Ghana: A nationwide survey using Patient Health Questionnaire-9 (PHQ-9)
Source: PLOS Ment Health. 2026 Jun 25;3(6):e0000498. doi: 10.1371/journal.pmen.0000498 (PMC13298969; doi:10.1371/journal.pmen.0000498)
Supplement: S2 Text — (DOCX) [file pmen.0000498.s003.docx]

## WHO STEPS Instrument

**
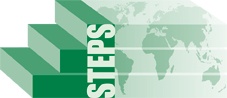
WHO STEPS Instrument**

**for Noncommunicable Disease
Risk Factor Surveillance**

**2023 Ghana**

| **Survey Information** |
| --- |

| **Location and Date** | | | **Response** | | **Code** | |
| --- | --- | --- | --- | --- | --- | --- |
| Cluster/Centre/Village ID | **└─┴─┴─┴─┴─┴─┘** | | I1 | |  |  |
| Cluster/Centre/Village name |  | | I2 | |  |  |
| Interviewer ID | └─┴─┴─┘ | | I3 | |  |  |
| Date of completion of the instrument | └─┴─┘ └─┴─┘ └─┴─┴─┴─┘  dd mm year | | I4 | |  |  |

| **Consent, Interview Language and Name** | **Response** | | **Code** |
| --- | --- | --- | --- |
| Consent has been read and obtained | Yes | 1 | I5 |
|  | No | 2 **If NO, END** |  |
| Interview Language *[English, TWI, Ga, Ewe, Dagbanli]* | English | 1 | I6 |
|  | *Twi* | 2 |  |
|  | *Ga* | 3 |  |
|  | *Ewe* | 4  Dagbanli 5 |  |
| Time of interview  (24-hour clock) | └─┴─┘: └─┴─┘  hrs mins | | I7 |
| Family Surname |  | | I8 |
| First Name |  | | I9 |
| **Additional Information that may be helpful** | | | |
| Contact phone number where possible |  | | I10 |

| **Step 1 Demographic Information** |
| --- |

| **CORE: Demographic Information** | | | | |
| --- | --- | --- | --- | --- |
| **Question** | **Response** | | | **Code** |
| Sex (*Record Male / Female as observed)* | Male | | 1 | C1 |
|  | Female | | 2 |  |
| What is your date of birth?  *Don't Know 77 77 7777* | └─┴─┘ └─┴─┘ └─┴─┴─┴─┘  dd mm year | | | C2 |
| How old are you? | Years | | └─┴─┘ | C3 |
| In total, how many years have you spent at school and in full-time study (excluding pre-school)? | Years | └─┴─┘ | | C4 |

| **EXPANDED: Demographic Information** | | | |
| --- | --- | --- | --- |
| What is the **highest level of education** you have completed? | No formal schooling | 1 | C5 |
|  | Pre-primary | 2 |  |
|  | Primary | 3 |  |
|  | Middle | 4 |  |
|  | Junior Secondary School/Junior High School | 5 |  |
|  | Secondary | 6 |  |
|  | Senior Secondary School /Senior High School  Higher | 7  8 |  |
|  | Refused/don’t know | 88 |  |
| What is your ethnic group? | Akan | 1 | C6 |
|  | Ga/Dangme | 2 |  |
|  | Ewe | 3 |  |
|  | Guan | 4 |  |
|  | Mole-Dagbani | 5 |  |
|  | Grusi | 6 |  |
|  | Gurma | 7 |  |
|  | Mande | 8 |  |
|  | Other (specify: ___________) | 9 |  |
|  | Refused | 88 |  |
| What is your religion? | Christian | 1 | X1 |
|  | Muslim | 2 |  |
|  | Traditionalist/Spiritual | 3 |  |
|  | None | 4 |  |
|  | Other (specify: ___________) | 9 |  |
|  | Refused | 88 |  |
|  |  |  |  |
| What is your **marital status**? | Never married | 1 | C7 |
|  | Currently married | 2 |  |
|  | Separated | 3 |  |
|  | Divorced | 4 |  |
|  | Widowed | 5 |  |
|  | Cohabitating | 6 |  |
|  | Refused | 88 |  |
| Which of the following best describes your **main** **work** status over the past 12 months?  *[INSERT COUNTRY-SPECIFIC CATEGORIES]*  (*USE SHOWCARD)* | Government employee | 1 | C8 |
|  | Non-government employee | 2 |  |
|  | Self-employed | 3 |  |
|  | Non-paid | 4 |  |
|  | Student | 5 |  |
|  | Housewife/Homemaker | 6 |  |
|  | Retired | 7 |  |
|  | Unemployed (able to work) | 8 |  |
|  | Unemployed (unable to work) | 9 |  |
|  | Refused | 88 |  |
| How many people older than 18 years, including yourself, live in your household? | Number of people | └─┴─┘ | C9 |
| TYPE OF TOILET FACILITY USUALLY USED BY THE HOUSEHOLD  OBSERVE AND RECORD | FLUSH OR POUR FLUSH TOILET  Flush to piped sewer system  Flush to septic tank  Flush to pit latrine  Flush to somewhere else  Flush, don't know where  PIT LATRINE  Ventilated improved pit latrine  Pit latrine with slab  Pit latrine without slab/ open pit  Bucket toilet  Hanging toilet/hanging latrine  No facility/bush/field  Other: (specify: ___________ ) | 1  2  3  4  5  6  7  8  9  10  11  12 | X2 |
| MAIN CONSTRUCTION MATERIAL USED FOR THE OUTER WALL  OBSERVE AND RECORD | Mud bricks/earth  Wood  Metal sheet/slate/asbestos  Stone  Burnt bricks  Cement blocks/concrete  Land Crete  Bamboo  Palm leaves/Thatch (grass/Raffia)  Other (Specify) | 1  2  3  4  5  6  7  8  9  10 | X3 |
| MAIN CONSTRUCTION MATERIAL USED FOR THE FLOOR  OBSERVE AND RECORD | Earth/Mud  Cement/Concrete  Stone  Burnt bricks  Wood  Vinyl tiles  Ceramic/Porcelain/Granite/Marble tiles  Terrazzo/Terrazzo tiles  Other (Specify) | 1  2  3  4  5  6  7  8  9 | X4 |
| MAIN MATERIAL OF THE ROOF  OBSERVE AND RECORD | NATURAL ROOFING  No roofing  Thatch/palm leaf  RUDIMENTARY ROOFING  Rustic mat  Palm/bamboo  Wood planks  Cardboard  FINISHED ROOFING  Wood  Calamine/cement fibre  Ceramic/brick tiles  Cement  Roofing Shingles  Asbestos/slate roofing sheets  Other (Specify) | 1  2  3  4  5  6  7  8  9  10  11  12  13 | x5 |
| Does your household have:  Furniture (stuffed)  Car/truck?  Bicycle?  Motor bike/scooter bike?  An animal-drawn cart?  A boat with a motor?  A boat without a motor?  Electricity?  Stove (kerosene)?  Stove (electric)?  Microwave?  Stove (gas)?  Satellite dish?  Washing machine?  Computer/Tablet computer?  Video deck/DVD/VCD?  A refrigerator?  A freezer?  Electric generator/Invertor(s)?  A land-line telephone?  Sewing machine?  A wall clock?  A radio?  A black/white television?  A colour television?  A mobile telephone?  Bed?  Table?  Cabinet/cupboard?  Access to the Internet in any device? | Furniture (stuffed)  Car/truck?  Bicycle?  Motor bike/scooter bike?  An animal-drawn cart?  A boat with a motor?  A boat without a motor?  Electricity?  Stove (kerosene)?  Stove (electric)?  Microwave?  Stove (gas)?  Satellite dish?  Washing machine?  Computer/Tablet computer?  Video deck/DVD/VCD?  A refrigerator?  A freezer?  Electric generator/Invertor(s)?  A land-line telephone?  Sewing machine?  A wall clock?  A radio?  A black/white television?  A colour television?  A mobile telephone?  Bed?  Table?  Cabinet/cupboard?  Access to the Internet in any device? | YES NO  1 2  1 2  1 2  1 2  1 2  1 2  1 2  1 2  1 2  1 2  1 2  1 2  1 2  1 2  1 2  1 2  1 2  1 2  1 2  1 2  1 2  1 2  1 2  1 2  1 2  1 2  1 2  1 2  1 2  1 2 | x6a  x6b  x6c  x6d  x6e  x6f  x6g  x6h  x6i  x6j  x6k  x6l  x6m  x6n  x6o  x6p  x6q  x6r  x6s  x6t  x6v  x6w  Cex6x  x6y  x6z  x6aa  x6ab  x6ac  x6ad  x6ae  x6af  x6ag  x6ah  x6ai  x6aj  Cex6ak  x6al  x6am |
| Does any member of the household own a house? | Yes  No | 1  2 | x7 |
| Does any member of the household own land/plot? | Yes  No | 1  2 | x8 |
| How many household members are covered by health insurance?  IF NONE, RECORD '00' | Number of people  Don't know/not sure | └─┴─┘    98 | X9 |

| **Step 1 Behavioural Measurements** |
| --- |

| **CORE: Tobacco Use** | | | |
| --- | --- | --- | --- |
| Now I am going to ask you some questions about tobacco use. | | | |
| **Question** | **Response** | | **Code** |
| Do you **currently** smoke any **tobacco** products, such as cigarettes, cigars or pipes?  *(USE SHOWCARD)* | Yes | 1 | T1 |
|  | No | 2 *If No, go to T8* |  |
| Do you currently smoke tobacco products **daily**? | Yes | 1 | T2 |
|  | No | 2 |  |
| How old were you when you **first started** smoking? | Age (years) | **└─┴─┘** *If Known, go to T5a/T5aw* | T3 |
|  | Don’t know 77 |  |  |
| Do you remember how long ago it was?  *(RECORD ONLY 1, NOT ALL 3)*  *Don’t know 77* | In Years | **└─┴─┘** *If Known, go to T5a/T5aw* | T4a |
|  | OR in Months | **└─┴─┘** *If Known, go to T5a/T5aw* | T4b |
|  | OR in Weeks | **└─┴─┘** | T4c |
| On average, **how many** of the following products do you smoke **each day/week?**  *(IF LESS THAN DAILY, RECORD WEEKLY)*  *(RECORD FOR EACH TYPE, USE SHOWCARD)*  *Don’t Know 7777* | DAILY**↓** WEEKLY↓ | | |
|  | Manufactured cigarettes | └─┴─┴─┴**─┘**└─┴─┴─┴**─┘** | T5a/T5aw |
|  | Hand-rolled cigarettes | └─┴─┴─┴**─┘**└─┴─┴─┴**─┘** | T5b/T5bw |
|  | Pipes full of tobacco | └─┴─┴─┴**─┘**└─┴─┴─┴**─┘** | T5c/T5cw |
|  | Cigars, cheroots, cigarillos | └─┴─┴─┴**─┘**└─┴─┴─┴**─┘** | T5d/T5dw |
|  | Number of Shisha sessions | └─┴─┴─┴**─┘**└─┴─┴─┴**─┘** | T5e/T5ew |
|  | Other | └─┴─┴─┴**─┘**└─┴─┴─┴**─┘** *If Other, go to T5other, else go to T6* | T5f/T5fw |
|  | Other (please specify): | └─┴─┴─┴─┴─┴─┘ | T5other/  T5otherw |
| During the past 12 months, have you tried to **stop smoking**? | Yes | 1 | T6 |
|  | No | 2 |  |
| During any visit to a doctor or other health worker in the past 12 months, were you advised to quit smoking tobacco? | Yes | 1 *If T2=Yes, go to T12; if T2=No, go* *to T9* | T7 |
|  | No | 2 *If T2=Yes, go to T12; if T2=No, go to T9* |  |
|  | No visit during the past 12 months | 3 *If T2=Yes, go to T12; if T2=No, go to T9* |  |
| In the past, did you **ever** **smoke** any tobacco products?  *(USE SHOWCARD)* | Yes | 1 | T8 |
|  | No | 2 *If No, go to T12* |  |
| In the past, did you **ever** smoke **daily**? | Yes | 1 *If T1=Yes, go to T12, else go to T10* | T9 |
|  | No | 2 *If T1=Yes, go to T12, else go to T10* |  |

| **EXPANDED: Tobacco Use** | | | |
| --- | --- | --- | --- |
| **Question** | **Response** | | **Code** |
| How old were you when you **stopped** smoking? | Age (years) | **└─┴─┘** *If Known, go to T12* | T10 |
|  | Don’t Know 77 |  |  |
| How **long ago** did you stop smoking?  *(RECORD ONLY 1, NOT ALL 3)*  *Don’t Know 77* | Years ago | **└─┴─┘** *If Known, go to T12* | T11a |
|  | OR Months ago | **└─┴─┘** *If Known, go to T12* | T11b |
|  | OR Weeks ago | **└─┴─┘** | T11c |
| Do you **currently use** any **smokeless tobacco** products such as *[snuff, chewing tobacco, betel]*? *(USE SHOWCARD)* | Yes | 1 | T12 |
|  | No | 2 *If No, go to T15* |  |
| Do you **currently use** **smokeless tobacco** products **daily?** | Yes | 1 | T13 |
|  | No | 2 *If No, go to T14aw* |  |
| On average, how many **times a day/week** do you use ….  *(IF LESS THAN DAILY, RECORD WEEKLY)*  *(RECORD FOR EACH TYPE, USE SHOWCARD)*  *Don’t Know 7777* | DAILY**↓** WEEKLY↓ | | |
|  | Snuff, by mouth | └─┴─┴─┴**─┘**└─┴─┴─┴**─┘** | T14a/  T14aw |
|  | Snuff, by nose | └─┴─┴─┴**─┘**└─┴─┴─┴**─┘** | T14b/  T14bw |
|  | Chewing tobacco | └─┴─┴─┴**─┘**└─┴─┴─┴**─┘** | T14c/  T14cw |
|  | Betel, quid | └─┴─┴─┴**─┘**└─┴─┴─┴**─┘** | T14d/  T14dw |
|  | Other | └─┴─┴─┴**─┘**└─┴─┴─┴**─┘** *If Other, go to T14other, if T13=No, go to T16, else go to T17* | T14e/  T14ew |
|  | Other (please specify): | └─┴─┴─┴─┴─┴─┘  *If T13=No, go to T16, else go to T17* | T14other/  T14otherw |
| In the **past**, did you **ever use** smokeless tobacco products such as *[snuff, chewing tobacco, or betel]*? | Yes | 1 | T15 |
|  | No | 2 *If No, go to T17* |  |
| In the **past**, did you **ever use** smokeless tobacco products such as *[snuff, chewing tobacco, or betel]* **daily**? | Yes | 1 | T16 |
|  | No | 2 |  |
| During the past 30 days, did someone smoke **in your home**? | Yes | 1 | T17 |
|  | No | 2 |  |
| During the past 30 days, did someone smoke in closed areas **in your workplace** (in the building, in a work area or a specific office)? | Yes | 1 | T18 |
|  | No | 2 |  |
|  | Don't work in a closed area | 3 |  |

| **CORE: Alcohol Consumption** | | | |
| --- | --- | --- | --- |
| The next questions ask about the consumption of alcohol. | | | |
| **Question** | **Response** | | **Code** |
| Have you **ever** consumed any alcohol such as beer, wine, spirits or pito, akpetsesi?  *(USE SHOWCARD OR SHOW EXAMPLES)* | Yes | 1 | A1 |
|  | No | 2  *If No, go to A16* |  |
| Have you consumed any alcohol within the **past 12 months**? | Yes | 1  *If Yes, go to A4* | A2 |
|  | No | 2 |  |
| Have you stopped drinking due to health reasons, such as a negative impact on your health or on the advice of your doctor or other health worker? | Yes | 1 *If Yes, go to A16* | A3 |
|  | No | 2 *If No, go to A16* |  |
| During the past 12 months, **how frequently** have you had at least one standard alcoholic drink?  *(READ RESPONSES, USE SHOWCARD)* | Daily | 1 | A4 |
|  | 5-6 days per week | 2 |  |
|  | 3-4 days per week | 3 |  |
|  | 1-2 days per week | 4 |  |
|  | 1-3 days per month | 5 |  |
|  | Less than once a month | 6 |  |
|  | Never | 7 |  |
| Have you consumed any alcohol within the **past 30 days**? | Yes | 1 | A5 |
|  | No | 2 *If No, go to A13* |  |
| During the past 30 days, on how many **occasions** did you have at least one standard alcoholic drink? | Number  Don't know 77 | └─┴─┘ *If Zero, go to A13* | A6 |
| During the past 30 days, when you drank alcohol, how many **standard** **drinks on average** did you have during one drinking occasion?  *(USE SHOWCARD)* | Number  Don't know 77 | └─┴─┘ | A7 |
| During the past 30 days, what was the **largest number** of standard drinks you had on a single occasion, counting all types of alcoholic drinks together? | Largest number  Don't Know 77 | └─┴─┘ | A8 |
| During the past 30 days, how many times did you have  **six or more** standard drinks in a single drinking occasion? | Number of times Don't Know 77 | └─┴─┘ | A9 |
| During each of the **past 7 days**, how many standard drinks did you have each day?  *(USE SHOWCARD)*  *Don't Know 77* | Monday | └─┴─┘ | A10a |
|  | Tuesday | └─┴─┘ | A10b |
|  | Wednesday | └─┴─┘ | A10c |
|  | Thursday | └─┴─┘ | A10d |
|  | Friday | └─┴─┘ | A10e |
|  | Saturday | └─┴─┘ | A10f |
|  | Sunday | └─┴─┘ | A10g |

| **CORE: Alcohol Consumption, continued** | | | |
| --- | --- | --- | --- |
| I have just asked you about your consumption of alcohol during the past 7 days. The questions were about alcohol in general, while the next questions refer to your consumption of homebrewed alcohol, alcohol brought over the border/from another country, any alcohol not intended for drinking or other untaxed alcohol. Please only think about these types of alcohol when answering the next questions. | | | |
| **Question** | **Response** | | **Code** |
| During the **past 7 days**, did you consume any **homebrewed** alcohol, any alcohol **brought over the border/from another country**, any alcohol **not intended for drinking** or other **untaxed** alcohol?  *[AMEND ACCORDING TO LOCAL CONTEXT]*  *(USE SHOWCARD)* | Yes | 1 | A11 |
|  | No | 2 *If No, go to A13* |  |
| On average, **how many standard drinks** of the following did you consume **during the past 7 days**?  *[INSERT COUNTRY-SPECIFIC EXAMPLES]*  *(USE SHOWCARD)*  *Don't Know 77* | Homebrewed spirits, e.g. Akpetsesi, Pito,  Atemuda, Shocker | **└─┴─┘** | A12a |
|  | Homebrewed beer or wine, e.g. beer, palm or fruit wine | **└─┴─┘** | A12b |
|  | Alcohol brought over the border/from another country | **└─┴─┘** | A12c |
|  | Alcohol not intended for drinking, e.g. alcohol-based medicines, perfumes, after shaves | **└─┴─┘** | A12d |
|  | Other untaxed alcohol in the country | **└─┴─┘** | A12e |

| **EXPANDED: Alcohol Consumption** | | | |
| --- | --- | --- | --- |
| During the **past 12 months,** how often have you found that you were not able to stop drinking once you had started? | Daily or almost daily | 1 | A13 |
|  | Weekly | 2 |  |
|  | Monthly | 3 |  |
|  | Less than monthly | 4 |  |
|  | Never | 5 |  |
| During the **past 12 months,** how often have you failed to do what was normally expected from you because of drinking? | Daily or almost daily | 1 | A14 |
|  | Weekly | 2 |  |
|  | Monthly | 3 |  |
|  | Less than monthly | 4 |  |
|  | Never | 5 |  |
| During the **past 12 months,** how often have you needed a first drink in the morning to get yourself going after a heavy drinking session? | Daily or almost daily | 1 | A15 |
|  | Weekly | 2 |  |
|  | Monthly | 3 |  |
|  | Less than monthly | 4 |  |
|  | Never | 5 |  |
| During the **past 12 months**, have you had family problems or problems with your partner due to **someone else’s** drinking? | Yes, more than monthly | 1 | A16 |
|  | Yes, monthly | 2 |  |
|  | Yes, several times but less than monthly | 3 |  |
|  | Yes, once or twice | 4 |  |
|  | No | 5 |  |

| **CORE: Diet** | | | | | | | |
| --- | --- | --- | --- | --- | --- | --- | --- |
| The next questions ask about the fruits and vegetables that you usually eat. I have a nutrition card here that shows you some examples of local fruits and vegetables. Each picture represents the size of a serving. As you answer these questions please think of a typical week in the last year. | | | | | | | |
| **Question** | | **Response** | | | | **Code** | |
| In a typical week, on how many days do you **eat fruit**?  *(USE SHOWCARD)* | | Number of days Don't Know 77 | *└─┴─┘ If Zero days, go to D3* | | | D1 | |
| How many **servings** of fruit do you eat on **one** of those days? (*USE SHOWCARD)* | | Number of servings  Don't Know 77 | └─┴─┘ | | | D2 | |
| In a typical week, on how many days do you **eat vegetables**? *(USE SHOWCARD)* | | Number of days Don't Know 77 | *└─┴─┘ If Zero days, go to D5* | | | D3 | |
| How many **servings** of vegetables do you eat on one of those days? *(USE SHOWCARD)* | | Number of servings  Don’t know 77 | └─┴─┘ | | | D4 | |
| **Dietary salt** | | | | | | | |
| With the next questions, we would like to learn more about salt in your diet. Dietary salt includes ordinary table salt, unrefined salt such as sea salt, iodized salt, salty stock cubes and powders, and salty sauces such as soy sauce or fish sauce (see showcard). The following questions are on adding salt to the food right before you eat it, on how food is prepared in your home, on eating processed foods that are high in salt such as *[insert country specific examples]*, and questions on controlling your salt intake. Please answer the questions even if you consider yourself to eat a diet low in salt. | | | | | | | |
| How often do you **add salt or a salty sauce such as soy sauce** to your food right before you eat it or as you are eating it?  *(SELECT ONLY ONE)*  *(USE SHOWCARD)* | | Always | | | 1 | D5 | |
|  |  | Often | | | 2 |  |  |
|  |  | Sometimes | | | 3 |  |  |
|  |  | Rarely | | | 4 |  |  |
|  |  | Never | | | 5 |  |  |
|  |  | Don't know | | | 77 |  |  |
| How often is **salt, salty seasoning or a salty sauce added** in cooking or preparing foods in your household? | | Always | | | 1 | D6 | |
|  |  | Often | | | 2 |  |  |
|  |  | Sometimes | | | 3 |  |  |
|  |  | Rarely | | | 4 |  |  |
|  |  | Never | | | 5 |  |  |
|  |  | Don't know | | | 77 |  |  |
| How often do you eat **processed food high in salt**? By processed food high in salt, I mean foods that have been altered from their natural state, such as packaged salty snacks, canned salty food including pickles and preserves, salty food prepared at a fast-food restaurant, cheese, bacon, processed meat, Koobi, Momoni, Kaako, salted pig feet*.*  *(USE SHOWCARD)* | | Always | | | 1 | D7 | |
|  |  | Often | | | 2 |  |  |
|  |  | Sometimes | | | 3 |  |  |
|  |  | Rarely | | | 4 |  |  |
|  |  | Never | | | 5 |  |  |
|  |  | Don't know | | | 77 |  |  |
| **How much salt or salty sauce** do you think you consume? | | Far too much | | | 1 | D8 | |
|  |  | Too much | | | 2 |  |  |
|  |  | Just the right amount | | | 3 |  |  |
|  |  | Too little | | | 4 |  |  |
|  |  | Far too little | | | 5 |  |  |
|  |  | Don't know | | | 77 |  |  |

| **EXPANDED: Diet** | | | | | | | | | | | |
| --- | --- | --- | --- | --- | --- | --- | --- | --- | --- | --- | --- |
| **Question** | | | **Response** | | | | | **Code** | | | |
| How important to you is **lowering the salt** in your diet? | | | | Very important | | | 1 | | D9 | | |
|  |  |  |  | Somewhat important | | | 2 | |  |  |  |
|  |  |  |  | Not at all important | | | 3 | |  |  |  |
|  |  |  |  | Don't know | | | 77 | |  |  |  |
| Do you think that too much salt or salty sauce in your diet could cause a **health problem**? | | | | Yes | | | 1 | | D10 | | |
|  |  |  |  | No | | | 2 | |  |  |  |
|  |  |  |  | Don't know | | | 77 | |  |  |  |
| Do you do any of the following on a regular basis to **control your salt intake**?  *(RECORD FOR EACH)* | | | | | | | | | | | |
| Limit consumption of processed foods | | | Yes | | | 1 | | D11a | | | |
|  |  |  | No | | | 2 | |  |  |  |  |
| Look at the salt or sodium content on food labels | | | Yes | | | 1 | | D11b | | | |
|  |  |  | No | | | 2 | |  |  |  |  |
| Buy low salt/sodium alternatives | | | Yes | | | 1 | | D11c | | | |
|  |  |  | No | | | 2 | |  |  |  |  |
| Use spices other than salt when cooking | | | Yes | | | 1 | | D11d | | | |
|  |  |  | No | | | 2 | |  |  |  |  |
| Avoid eating foods prepared outside of a home | | | Yes | | | 1 | | D11e | | | |
|  |  |  | No | | | 2 | |  |  |  |  |
| Do other things specifically to control your salt intake | | | Yes | | | 1  *If Yes, go to D11other* | | D11f | | | |
|  |  |  | No | | | 2 | |  |  |  |  |
| Other (please specify) | | | └─┴─┴─┴─┴─┴─┴─┘ | | | | | D11other | | | |

| **CORE: Physical Activity** | | | |
| --- | --- | --- | --- |
| Next I am going to ask you about the time you spend doing different types of physical activity in a typical week. Please answer these questions even if you do not consider yourself to be a physically active person.  Think first about the time you spend doing work. Think of work as the things that you have to do such as paid or unpaid work, study/training, household chores, harvesting food/crops, fishing or hunting for food, seeking employment. *[Insert other examples if needed].* In answering the following questions 'vigorous-intensity activities' are activities that require hard physical effort and cause large increases in breathing or heart rate, 'moderate-intensity activities' are activities that require moderate physical effort and cause small increases in breathing or heart rate. | | | |
| **Question** | **Response** | | **Code** |
| **Work** | | | |
| Does your work involve vigorous-intensity activity that causes large increases in breathing or heart rate like *[carrying or lifting* *heavy loads, digging or construction work*?  *[INSERT EXAMPLES] (USE SHOWCARD)* | Yes | 1 | P1 |
|  | No | 2  *If No, go to P 4* |  |
| In a typical week, on how many days do you do vigorous-intensity activities as part of your work? | Number of days | └─┘ | P2 |
| How much time do you spend doing vigorous-intensity activities at work on a typical day? | Hours: minutes | └─┴─┘: └─┴─┘  hrs mins | P3 (a-b) |
| Does your work involve moderate-intensity activity, that causes small increases in breathing or heart rate such as brisk walking *[or carrying light loads*?  *[INSERT EXAMPLES] (USE SHOWCARD)* | Yes | 1 | P4 |
|  | No | 2 *If No, go to P 7* |  |
| In a typical week, on how many days do you do moderate-intensity activities as part of your work? | Number of days | └─┘ | P5 |
| How much time do you spend doing moderate-intensity activities at work on a typical day? | Hours: minutes | └─┴─┘: └─┴─┘  hrs mins | P6 (a-b) |
| **Travel to and from places** | | | |
| The next questions exclude the physical activities at work that you have already mentioned.  Now I would like to ask you about the usual way you travel to and from places. For example to work, for shopping, to market, to place of worship. *[Insert other examples if needed]* | | | |
| Do you walk or use a bicycle *(pedal cycle* to get to and from places? | Yes | 1 | P7 |
|  | No | 2  *If No, go to P 10* |  |
| In a typical week, on how many days do you walk or bicycle to get to and from places? | Number of days | └─┘ | P8 |
| How much time do you spend walking or bicycling for travel on a typical day? | Hours: minutes | └─┴─┘: └─┴─┘  hrs mins | P9 (a-b) |

| **CORE: Physical Activity, Continued** | | | |
| --- | --- | --- | --- |
| **Question** | **Response** | | **Code** |
| **Recreational activities** | | | |
| The next questions exclude the work and transport activities that you have already mentioned.  Now I would like to ask you about sports, fitness and recreational activities (leisure), *[Insert relevant terms]*. | | | |
| Do you do any vigorous-intensity sports, fitness or recreational *(leisure)* activities that cause large increases in breathing or heart rate like *[running or football*?  *[INSERT EXAMPLES] (USE SHOWCARD)* | Yes | 1 | P10 |
|  | No | 2  *If No, go to P 13* |  |
| In a typical week, on how many days do you do vigorous-intensity sports, fitness or recreational *(leisure)* activities? | Number of days | └─┘ | P11 |
| How much time do you spend doing vigorous-intensity sports, fitness or recreational activities on a typical day? | Hours: minutes | └─┴─┘: └─┴─┘  hrs mins | P12  (a-b) |
| Do you do any moderate-intensity sports, fitness or recreational *(leisure)* activities that cause a small increase in breathing or heart rate such as brisk walking*, [cycling, swimming, volleyball]*  *[INSERT EXAMPLES] (USE SHOWCARD)* | Yes | 1 | P13 |
|  | No | 2  *If No, go to P16* |  |
| In a typical week, on how many days do you do moderate-intensity sports, fitness or recreational *(leisure)* activities? | Number of days | └─┘ | P14 |
| How much time do you spend doing moderate-intensity sports, fitness or recreational *(leisure)* activities on a typical day? | Hours: minutes | └─┴─┘: └─┴─┘  hrs mins | P15 (a-b) |

| **EXPANDED: Physical Activity** | | | |
| --- | --- | --- | --- |
| **Sedentary behaviour** | | | |
| The following question is about sitting or reclining at work, at home, getting to and from places, or with friends including time spent sitting at a desk, sitting with friends, traveling in car, bus, train, reading, playing cards or watching television, but do not include time spent sleeping.  *[INSERT EXAMPLES] (USE SHOWCARD)* | | | |
| How much time do you usually spend sitting or reclining on a typical day? | Hours: minutes | └─┴─┘: └─┴─┘  hrs mins | P16  (a-b) |

| **CORE: History of Elevated Blood Pressure** | | | |
| --- | --- | --- | --- |
| **Question** | **Response** | | **Code** |
| Have you ever had your blood pressure measured by a doctor or other health worker? | Yes | 1 | H1 |
|  | No | 2 *If No, go to H6* |  |
| Have you ever been told by a doctor or other health worker that you have elevated blood pressure or hypertension? | Yes | 1 | H2a |
|  | No | 2  *If No, go to H6* |  |
| Were you first told in the past 12 months? | Yes | 1 | H2b |
|  | No | 2 |  |
| In the past two weeks, have you taken any drugs (medication) for elevated blood pressure prescribed by a doctor or other health worker? | Yes | 1 | H3 |
|  | No | 2 |  |
| Have you ever seen a traditional healer for elevated blood pressure or hypertension? | Yes | 1 | H4 |
|  | No | 2 |  |
| Are you currently taking any herbal or traditional remedy for your elevated blood pressure? | Yes | 1 | H5 |
|  | No | 2 |  |

| **CORE: History of Diabetes** | | | |
| --- | --- | --- | --- |
| Have you ever had your blood sugar measured by a doctor or other health worker? | Yes | 1 | H6 |
|  | No | 2 *If No, go to H12* |  |
| Have you ever been told by a doctor or other health worker that you have elevated blood sugar or diabetes? | Yes | 1 | H7a |
|  | No | 2  *If No, go to H12* |  |
| Were you first told in the past 12 months? | Yes | 1 | H7b |
|  | No | 2 |  |
| In the past two weeks, have you taken any drugs (medication) for diabetes prescribed by a doctor or other health worker? | Yes | 1 | H8 |
|  | No | 2 |  |
| Are you currently taking insulin for diabetes prescribed by a doctor or other health worker? | Yes | 1 | H9 |
|  | No | 2 |  |
| Have you ever seen a traditional healer for diabetes or elevated blood sugar? | Yes | 1 | H10 |
|  | No | 2 |  |
| Are you currently taking any herbal or traditional remedy for your diabetes? | Yes | 1 | H11 |
|  | No | 2 |  |

| **CORE: History of Elevated Total Cholesterol** | | | | | | |  |
| --- | --- | --- | --- | --- | --- | --- | --- |
| **Question** | | **Response** | | | **Code** | | |
| Have you ever had your cholesterol (fat levels in your blood) measured by a doctor or other health worker? | | Yes | 1 | | | H12 | |
|  |  | No | 2 *If No, go to H17* | | |  |  |
| Have you ever been told by a doctor or other health worker that you have elevated cholesterol? | | Yes | 1 | | | H13a | |
|  |  | No | 2 *If No, go to H17* | | |  |  |
| Were you first told in the past 12 months? | | Yes | 1 | | | H13b | |
|  |  | No | 2 | | |  |  |
| In the past two weeks, have you taken any oral treatment (medication) for elevated total cholesterol prescribed by a doctor or other health worker? | | Yes | 1 | | | H14 | |
|  |  | No | 2 | | |  |  |
| Have you ever seen a traditional healer for elevated cholesterol? | | Yes | 1 | | | H15 | |
|  |  | No | 2 | | |  |  |
| Are you currently taking any herbal or traditional remedy for your elevated cholesterol? | | Yes | 1 | | | H16 | |
|  |  | No | 2 | | |  |  |

| **CORE: History of Cardiovascular Diseases** | | | |
| --- | --- | --- | --- |
| Have you ever had a heart attack or chest pain from heart disease (angina) or a stroke (cerebrovascular accident or incident)?  USE SHOW CARD DEPICTING LOCATION OF SHOW CARD | Yes | 1 | H17 |
|  | No | 2 |  |
| Are you currently taking aspirin regularly to prevent or treat heart disease? | Yes | 1 | H18 |
|  | No | 2 |  |
| Are you currently taking statins (cholesterol lowering medications (Lovastatin/Simvastatin/Atorvastatin or any other statin) regularly to prevent or treat heart disease? | Yes | 1 | H19 |
|  | No | 2 |  |

| **CORE: Lifestyle Advice** | | | | |
| --- | --- | --- | --- | --- |
| **Question** | | **Response** | | **Code** |
| During the past 12 months, have you visited a doctor or other health worker? | | Yes | 1 | H20 |
|  |  | No | 2 *If No and C1=1, go to MH1*  *If No and C1=2, go to CX1* |  |
| During any of your visits to a doctor or other health worker in the past 12 months, were you advised to do any of the following?  *(RECORD FOR EACH)* | | | | |
| Quit using tobacco or don’t start | | Yes | 1 | H20a |
|  |  | No | 2 |  |
| Reduce salt in your diet | | Yes | 1 | H20b |
|  |  | No | 2 |  |
| Eat at least five servings of fruit and/or vegetables each day | | Yes | 1 | H20c |
|  |  | No | 2 |  |
| Reduce fat in your diet | | Yes | 1 | H20d |
|  |  | No | 2 |  |
| Start or do more physical activity | | Yes | 1 | H20e |
|  |  | No | 2 |  |
| Maintain a healthy body weight or lose weight | | Yes | 1 | H20f |
|  |  | No | 2 |  |
| Reduce sugary beverages in your diet | | Yes | 1  *If C1=1 go to MH1* | H20g |
|  |  | No | 2 |  |

| **Cervical Cancer** | | | | | | | | | | | |
| --- | --- | --- | --- | --- | --- | --- | --- | --- | --- | --- | --- |
| **CORE (for women only): Cervical Screening** | | | | | | | | |  |  |  |
| **The next questions ask about cervical prevention.**  **Screening tests for cervical cancer prevention can be done in different ways, including Visual Inspection with Acetic Acid/vinegar (VIA), pap smear and Human Papillomavirus (HPV) test. VIA is an inspection of the surface of the uterine cervix after acetic acid (or vinegar) has been applied to it. For both pap smear and HPV test, a doctor or nurse uses a swab to wipe from inside your vagina, take a sample and send it to a laboratory. It is even possible that you were given the swab yourself and asked to swab the inside of your vagina. The laboratory checks for abnormal cell changes if a pap smear is done, and for the HP virus if an HPV test is done.** | | | | | | | | |  |  |  |
| The next questions ask about cervical cancer prevention. Screening tests for cervical cancer prevention can be done in different ways, including Visual Inspection with Acetic Acid/vinegar (VIA), pap smear and Human Papillomavirus (HPV) test. VIA is an inspection of the surface of the uterine cervix after acetic acid (or vinegar) has been applied to it. For both pap smear and HPV test, a doctor or nurse uses a swab to wipe from inside your vagina, take a sample and send it to a laboratory. It is even possible that you were given the swab yourself and asked to swab the inside of your vagina. The laboratory checks for abnormal cell changes if a pap smear is done, and for the HP virus if an HPV test is done. | | | | | | | | | | | |
| **Question** | | | **Response** | | | |  | | **Code** | | |
| Have you ever had a screening test for cervical cancer, using any of these methods described above? | | | Yes | | | | 1 | | CX1 | | |
|  |  |  | No | | | | 2 | |  |  |  |
|  |  |  | Don’t know | | | | 77 | |  |  |  |
| If Yes, please indicate the type pf test that was carried out | | | HPV-DNA | | | | 1 | | CXX1 | | |
|  | | | Pap Smear | | | | 2 | |  | | |
|  | | | Visual Inspection with Acetic Acid | | | | 3 | |  | | |
| *The next questions CX2 – CX10 are administered only to those that ever had a screening test for cervical cancer (CX1=1). If CX1=2, go to CX11.* | | | | | | | | |  |  |  |
| At what age were you **first tested** for cervical cancer? | Age | | └─┴─┘ | | | | CX2 | |  |  |  |
|  | Don't know | | 77 | | | |  |  |  |  |  |
|  | Refused | | 88 | | | |  |  |  |  |  |
| When was your **last (most recent) test** for cervical cancer? | Less than 1 year ago | | 1 | | | | CX3 | |  |  |  |
|  | 1-2 years ago | | 2 | | | |  |  |  |  |  |
|  | 3-5 years ago | | 3 | | | |  |  |  |  |  |
|  | More than 5 years ago | | 4 | | | |  |  |  |  |  |
|  | Don't know | | 77 | | | |  |  |  |  |  |
|  | Refused | | 88 | | | |  |  |  |  |  |
| What is the **main reason** you had your **last** test for cervical cancer? | Part of a routine exam | | 1 | | | | CX4 | |  |  |  |
|  | Following up on abnormal or inconclusive result | | 2 | | | |  |  |  |  |  |
|  | Recommended by healthcare provider | | 3 | | | |  |  |  |  |  |
|  | Recommended by other source | | 4 | | | |  |  |  |  |  |
|  | Experiencing pain or other symptoms | | 5 | | | |  |  |  |  |  |
|  | Other | | 6 | | | |  |  |  |  |  |
|  | Don't know | | | 77 | | |  |  |  |  |  |
|  | Refused | | | 88 | | |  |  |  |  |  |
| **Where** did you receive your last test for cervical cancer?  *[INSERT COUNTRY-SPECIFIC CATEGORIES]* | Doctor’s office | | 1 | | | | CX5 | |  |  |  |
|  | Mobile clinic | | 2 | | | |  |  |  |  |  |
|  | Community clinic | | 3 | | | |  |  |  |  |  |
|  | Hospital | | 4 | | | |  |  |  |  |  |
|  | Other | | 5 | | | |  |  |  |  |  |
|  | Don't know | | 77 | | | |  |  |  |  |  |
|  | Refused | | 88 | | | |  |  |  |  |  |
| What was the result of your **last (most recent)** test for cervical cancer? | Did not receive result | | | | 1 *If CC6=1, go to next section* | | CX6 | |  |  |  |
|  | Normal / Negative | | | | 2 *If CC6=2, go to next section* | |  |  |  |  |  |
|  | Abnormal /Positive | | | | 3 | |  |  |  |  |  |
|  | Suspect cancer | | | | 4 | |  |  |  |  |  |
|  | Inconclusive | | | | 5 | |  |  |  |  |  |
|  | Don’t know | | | | 77 | |  |  |  |  |  |
|  | Refused | | | | 88 | |  |  |  |  |  |
| Did you have any follow-up visits because of your test results? | Yes | | | | 1 | | CX7 | |  |  |  |
|  | No | | | | 2 | |  |  |  |  |  |
|  | Don't know | | | | 77 | |  |  |  |  |  |
|  | Refused | | | | 88 | |  |  |  |  |  |
| Did you receive any treatment to your cervix because of your test result? | Yes | | | | 1 | | CX8 | |  |  |  |
|  | No | | | | 2 *If No, go to CC10* | |  |  |  |  |  |
|  | Don't know | | | | 77 *If Don’t know, go to*  *next section* | |  |  |  |  |  |
|  | Refused | | | | 88 *If Refused, go to*  *next section* | |  |  |  |  |  |
| Did you receive treatment **during the same visit** as your last test for cervical cancer? | Yes | | | | 1 *If Yes, go to next section* | | CX9 | |  |  |  |
|  | No | | | | 2 *If No, go to next section* | |  |  |  |  |  |
|  | Don't know | | | | 77 *If Don’t know, go to*  *next section* | |  |  |  |  |  |
|  | Refused | | | | 88 *If Refused, go to*  *next section* | |  |  |  |  |  |
| What is the **main** reason you did not receive treatment? | Was not told I needed treatment | | | | 1 | | CX10 | |  |  |  |
|  | Did not know how/where to get treatment | | | | 2 | |  |  |  |  |  |
|  | Embarrassment | | | | 3 | |  |  |  |  |  |
|  | Too expensive | | | | 4 | |  |  |  |  |  |
|  | Didn’t have time | | | | 5 | |  |  |  |  |  |
|  | Clinic too far away | | | | 6 | |  |  |  |  |  |
|  | Poor service quality | | | | 7 | |  |  |  |  |  |
|  | Fear (afraid of procedure; afraid of social stigma) | | | | 8 | |  |  |  |  |  |
|  | Cultural beliefs | | | | 9 | |  |  |  |  |  |
|  | Family member would not allow it | | | | 10  *If CC10=10, go to C10Spec,*  *else go to next section* | |  |  |  |  |  |
|  | Don’t know | | | | 77 | |  |  |  |  |  |
|  | Refused | | | | 88 | |  |  |  |  |  |
|  | Family member (please specify) | | | | └─┴─┴─┴─┴─┴─┴─┘ | | CX10Spec | |  |  |  |
| What is the **main** reason you have never had a cervical cancer test? | Did not know how/where to get test | | | | 1 | | CX11 | |  |  |  |
|  | Embarrassment | | | | 2 | |  |  |  |  |  |
|  | Too expensive | | | | 3 | |  |  |  |  |  |
|  | Didn’t have time | | | | 4 | |  |  |  |  |  |
|  | Clinic too far away | | | | 5 | |  |  |  |  |  |
|  | Poor service quality | | | | 6 | |  |  |  |  |  |
|  | Fear (afraid of procedure; afraid of social stigma) | | | | 7 | |  |  |  |  |  |
|  | Cultural beliefs | | | | 8 | |  |  |  |  |  |
|  | Family member would not allow it | | | | 9  *If CC11=9, go to C11Spec,*  *else go to next section* | |  |  |  |  |  |
|  | Don’t know | | | | 77 | |  |  |  |  |  |
|  | Refused | | | | 88 | |  |  |  |  |  |
|  | Family member (please specify) | | | | └─┴─┴─┴─┴─┴─┴─┘ | | CX11Spec | |  |  |  |

| **Addition (for women only): Breast Cancer Screening** | | | | | | | | | | | |  |  |
| --- | --- | --- | --- | --- | --- | --- | --- | --- | --- | --- | --- | --- | --- |
| The next questions ask about breast cancer prevention. Screening tests for breast cancer prevention can be done to identify breast cancer before any symptoms appear. There are various screening methods for breast cancer, such as mammography and clinical breast exam. For mammography, X-rays are used to identify abnormal tissues, while clinical breast exam is physical examination of both breasts by a trained health professional. | | | | | | | | | | | |  |  |
| Have you ever had a screening test for breast cancer, using any of these methods described above? | | Yes | | | | | | 1 | | | BX1 |  |  |
|  |  | No | | | | | | 2 | | |  |  |  |
|  |  | Don’t know | | | | | | 77 | | |  |  |  |
| *Which type of test did you receive Mammography 1 X-Ray 3*  *Clinical Breast Examination 2* | | | | *Mammography* | | | | *1* | | | *BX2* | | |
|  | | | | *Clinical breast Examination* | | | | *2* | | |  |  |  |
|  | | | | *X-Ray* | | | | *3* | | |  |  |  |
| At what age were you **first screened/ tested** for breast cancer? | | Age | | | └─┴─┘ | | | | | BX3 | | | |
|  |  | Don't know | | | 77 | | | | |  |  |  |  |
|  |  | Refused | | | 88 | | | | |  |  |  |  |
| When was your **last (most recent) screen/test** for breast cancer? | | Less than 1 year ago | | | 1 | | | | | BX4 | | | |
|  |  | 1-2 years ago | | | 2 | | | | |  |  |  |  |
|  |  | 3-5 years ago | | | 3 | | | | |  |  |  |  |
|  |  | More than 5 years ago | | | 4 | | | | |  |  |  |  |
|  |  | Don't know | | | 77 | | | | |  |  |  |  |
|  |  | Refused | | | 88 | | | | |  |  |  |  |
| What is the **main reason** you had your **last** screening/test for breast cancer? | | Part of a routine exam | | | 1 | | | | | BX5 | | | |
|  |  | Following up on abnormal or inconclusive result | | | 2 | | | | |  |  |  |  |
|  |  | Recommended by healthcare provider | | | 3 | | | | |  |  |  |  |
|  |  | Recommended by other source | | | 4 | | | | |  |  |  |  |
|  |  | Experienced pain or other symptoms such as lumps | | | 5 | | | | |  |  |  |  |
|  |  | Other | | | 6 | | | | |  |  |  |  |
|  |  | Don't know | | | | 77 | | | |  |  |  |  |
|  |  | Refused | | | | 88 | | | |  |  |  |  |
| **Where** did you receive your last screening/test for breast cancer?  *[INSERT COUNTRY-SPECIFIC CATEGORIES]* | | Doctor’s office | | | 1 | | | | | BX6 | | | |
|  |  | Mobile clinic | | | 2 | | | | |  |  |  |  |
|  |  | Community clinic | | | 3 | | | | |  |  |  |  |
|  |  | Hospital | | | 4 | | | | |  |  |  |  |
|  |  | Other | | | 5 | | | | |  |  |  |  |
|  |  | Don't know | | | 77 | | | | |  |  |  |  |
|  |  | Refused | | | 88 | | | | |  |  |  |  |
| What was the result of your **last (most recent)** test for breast cancer? | | Did not receive result | | | | | 1 *If BX6=1, go to next section* | | | BX7 | | | |
|  |  | Normal / Negative | | | | | 2 *If BX6=2, go to next section* | | |  |  |  |  |
|  |  | Abnormal /Positive | | | | | 3 | | |  |  |  |  |
|  |  | Suspect cancer | | | | | 4 | | |  |  |  |  |
|  |  | Inconclusive | | | | | 5 | | |  |  |  |  |
|  |  | Don’t know | | | | | 77 | | |  |  |  |  |
|  |  | Refused | | | | | 88 | | |  |  |  |  |
| Did you have any follow-up visits because of your breast screening/test results? | | Yes | | | | | 1 | | | BX8 | | | |
|  |  | No | | | | | 2 | | |  |  |  |  |
|  |  | Don't know | | | | | 77 | | |  |  |  |  |
|  |  | Refused | | | | | 88 | | |  |  |  |  |
| Did you receive any treatment/surgical procedures to your breast(s) because of your screening/test result? | | Yes | | | | | 1 *If Yes, go to next section* | | | BX9 | | | |
|  |  | No | | | | | 2 | | |  |  |  |  |
|  |  | Don't know | | | | | 77 *If Don’t know, go to*  *next section* | | |  |  |  |  |
|  |  | Refused | | | | | 88 *If refused, go to*  *next section* | | |  |  |  |  |
| What is the **main** reason you did not receive treatment? | | Was not told I needed treatment | | | | | 1 | | | BX10 | | | |
|  |  | Did not know how/where to get treatment | | | | | 2 | | |  |  |  |  |
|  |  | Embarrassment | | | | | 3 | | |  |  |  |  |
|  |  | Too expensive | | | | | 4 | | |  |  |  |  |
|  |  | Didn’t have time | | | | | 5 | | |  |  |  |  |
|  |  | Clinic too far away | | | | | 6 | | |  |  |  |  |
|  |  | Poor service quality | | | | | 7 | | |  |  |  |  |
|  |  | Fear (afraid of procedure; afraid of social stigma) | | | | | 8 | | |  |  |  |  |
|  |  | Cultural beliefs | | | | | 9 | | |  |  |  |  |
|  |  | Family member would not allow it | | | | | 10  *If BX10=10, go to BX10Spec,*  *else go to next section* | | |  |  |  |  |
|  |  | Don’t know | | | | | 77 | | |  |  |  |  |
|  |  | Refused | | | | | 88 | | |  |  |  |  |
|  |  | Family member (please specify) | | | | | └─┴─┴─┴─┴─┴─┴─┘ | | | BX10Spec | | | |
| What is the **main** reason you have never had a breast cancer screening/test? | | Did not know how/where to get test | | | | | 1 | | | BX11 | | | |
|  |  | Embarrassment | | | | | 2 | | |  |  |  |  |
|  |  | Too expensive | | | | | 3 | | |  |  |  |  |
|  |  | Didn’t have time | | | | | 4 | | |  |  |  |  |
|  |  | Clinic too far away | | | | | 5 | | |  |  |  |  |
|  |  | Poor service quality | | | | | 6 | | |  |  |  |  |
|  |  | Fear (afraid of procedure; afraid of social stigma) | | | | | 7 | | |  |  |  |  |
|  |  | Cultural beliefs | | | | | 8 | | |  |  |  |  |
|  |  | Family member would not allow it | | | | | 9  *If BX10=9, go to BX10Spec,*  *else go to next section* | | |  |  |  |  |
|  |  | Don’t know | | | | | 77 | | |  |  |  |  |
|  |  | Refused | | | | | 88 | | |  |  |  |  |
|  |  | Family member (please specify) | | | | | └─┴─┴─┴─┴─┴─┴─┘ | | | CX11Spec | | | |

| **Mental health / Depression/Suicide** |
| --- |

| **Mental health / Suicide** | | | |
| --- | --- | --- | --- |
| The next questions ask about thoughts, plans, and attempts of suicide. Please answer the questions even if no one usually talks about these issues. | | | |
| **Question** | **Response** | | **Code** |
| What do you think causes mental health conditions? | DISEASE  CURSE/SPELL  OTHER (SPECIFY: __________________)  DON’T KNOW  REFUSED | 1  2  3  99  88 | ExMH01 |
| Are mental health conditions treatable? | YES  NO  DON’T KNOW  REFUSSED | 1  2  99  88 | ExMH02 |
| Have you ever been diagnosed as having a mental health condition? | YES  NO  REFUSED | 1  2 Go to ExMH05  88 Go to ExMH05 | ExMH03 |
| If yes, what condition(s) were you diagnosed as having? | Depression  Mood Disorders (Anxiety, Bipolar)  Schizophrenia | 1  2  3 | ExMH04 |
| During the last two-week period, did you feel sad or empty every day, nearly every day, most days, about half the days or less than half the days? Specify. | NOT AT ALL  SEVERAL DAYS  MORE THAN HALF THE DAYS  NEARLY EVERYDAY  REFUSSED | 4  3  2  1  88 | ExMH05 |
| During the last two-week period, did you feel little interest or pleasure in doing things | NOT AT ALL  SEVERAL DAYS  MORE THAN HALF THE DAYS  NEARLY EVERYDAY  REFUSSED | 4  3  2  1  88 | ExMH06 |
| During the two-week period, did you feel down, depressed, or hopeless | NOT AT ALL  SEVERAL DAYS  MORE THAN HALF THE DAYS  NEARLY EVERYDAY  REFUSSED | 4  3  2  1  88 | ExMH07 |
| During the last two weeks, did you have trouble falling or staying asleep, or sleeping too much | NOT AT ALL  SEVERAL DAYS  MORE THAN HALF THE DAYS  NEARLY EVERYDAY  REFUSSED | 4  3  2  1  88 | ExMH08 |
| During the last two weeks did you experience of feeling tired or having little energy | NOT AT ALL  SEVERAL DAYS  MORE THAN HALF THE DAYS  NEARLY EVERYDAY  REFUSSED | 4  3  2  1  88 | ExMH9 |
| During the last two weeks did you, did you experience poor appetite or overeating | NOT AT ALL  SEVERAL DAYS  MORE THAN HALF THE DAYS  NEARLY EVERYDAY  REFUSSED | 4  3  2  1  88 | ExMH10 |
| During the last two weeks, did you have a feel bad about yourself, or that you are a failure, or have let yourself or your family down | NOT AT ALL  SEVERAL DAYS  MORE THAN HALF THE DAYS  NEARLY EVERYDAY  REFUSSED | 4  3  2  1  88 | ExMH11 |
| During the last two weeks, did you have trouble concentrating on things | NOT AT ALL  SEVERAL DAYS  MORE THAN HALF THE DAYS  NEARLY EVERYDAY  REFUSSED | 4  3  2  1  88 | ExMH12 |
| During the last two weeks, did you experience moving or speaking so slowly that other people could have noticed. Or the opposite – being so fidgety or restless that you have been moving around a lot more than usual. | NOT AT ALL  SEVERAL DAYS  MORE THAN HALF THE DAYS  NEARLY EVERYDAY  REFUSSED | 4  3  2  1  88 | ExMH13 |
| During the last two weeks, did you attempt suicide or feel like committing suicide | NOT AT ALL  SEVERAL DAYS  MORE THAN HALF THE DAYS  NEARLY EVERYDAY  REFUSSED | 4  3  2  1  88 | ExMH14 |
| Did people complain about your change in behaviour? | YES  NO  REFUSED | 1  2  88 | ExMH15 |
| During the **past 12 months**, have you seriously **considered** attempting suicide? | Yes | 1 | MH1 |
|  | No | 2 *If No, go to MH3* |  |
|  | Refused | 88 |  |
| Did you seek **professional help** for these thoughts? | Yes | 1 | MH2 |
|  | No | 2 |  |
|  | Refused | 88 |  |
| During the **past 12 months**, have you made **a plan about how** you would attempt suicide? | Yes | 1 | MH3 |
|  | No | 2 |  |
|  | Refused | 88 |  |
| Have you **ever attempted suicide**? | Yes | 1 | MH4 |
|  | No | 2 *If No, go to MH9* |  |
|  | Refused | 88 |  |
| During the **past 12 months**, have you **attempted suicide**? | Yes | 1 | MH5 |
|  | No | 2 |  |
|  | Refused | 88 |  |
| What was the main **method you used** the last time you attempted suicide?  *(SELECT ONLY ONE)* | Razor, knife or another sharp instrument | 1 | MH6 |
|  | Overdose of medication (e. g. prescribed, over-the-counter) | 2 |  |
|  | Overdose of other substance (e.g. heroin, crack, alcohol) | 3 |  |
|  | Poisoning with pesticides (e.g. rat poison, insecticide, ddt) | 4 |  |
|  | Other poisoning (e.g. plant/seed, household product) | 5 |  |
|  | Poisonous gases from exhaust | 6 |  |
|  | Other | 7 *If Other, go to MH6other* |  |
|  | Refused | 88 |  |
|  | Other (specify) | ______________________ | MH6other |
| Did you seek **medical care** for this attempt? | Yes | 1 | MH7 |
|  | No | 2 *If No, go to MH9* |  |
|  | Refused | 88 |  |
| Were you **admitted to hospital overnight** because of this attempt? | Yes | 1 | MH8 |
|  | No | 2 |  |
|  | Refused | 88 |  |
| Has anyone in **your close family** (mother, father, brother, sister or children) ever attempted suicide? | Yes | 1 | MH9 |
|  | No | 2 |  |
|  | Refused | 88 |  |
| Has anyone in **your close family** (mother, father, brother, sister or children) ever died from suicide? | Yes | 1 | MH10 |
|  | No | 2 |  |
|  | Refused | 88 |  |

| **Oral Health** |
| --- |

| **Oral Health** | | | |
| --- | --- | --- | --- |
| The next questions ask about your oral health status and related behaviours. | | | |
| **Question** | **Response** | | **Code** |
| How many **natural teeth** do you have? | No natural teeth | 1  *If No natural teeth, go to O4* | O1 |
|  | 1 to 9 teeth | 2 |  |
|  | 10 to 19 teeth | 3 |  |
|  | 20 teeth or more | 4 |  |
|  | Don't know | 77 |  |
| How would you describe the **state of your teeth?** | Excellent | 1 | O2 |
|  | Very Good | 2 |  |
|  | Good | 3 |  |
|  | Average | 4 |  |
|  | Poor | 5 |  |
|  | Very Poor | 6 |  |
|  | Don't Know | 77 |  |
| How would you describe the **state of your gums?** | Excellent | 1 | O3 |
|  | Very Good | 2 |  |
|  | Good | 3 |  |
|  | Average | 4 |  |
|  | Poor | 5 |  |
|  | Very Poor | 6 |  |
|  | Don't know | 77 |  |
| How would you describe the **state of your mouth (mucosa)?** | Excellent | 1 | O4 |
|  | Very Good | 2 |  |
|  | Good | 3 |  |
|  | Average | 4 |  |
|  | Poor | 5 |  |
|  | Very Poor | 6 |  |
|  | Don't know | 77 |  |
| Do you have any **removable dentures**? | Yes | 1 | O5 |
|  | No | 2 *If No, go to O7* |  |
| Which of the following removable dentures do you have?  *(RECORD FOR EACH)* | | |  |
| An upper jaw denture | Yes | 1 | O6a |
|  | No | 2 |  |
| A lower jaw denture | Yes | 1 | O6b |
|  | No | 2 |  |
| During the past 12 months, did your teeth, gums or mouth cause any **pain or discomfort**? | Yes | 1 | O7 |
|  | No | 2 |  |
| How long has it been since you last **saw a dentist**? | Less than 6 months | 1 | O8 |
|  | 6-12 months | 2 |  |
|  | More than 1 year but less than 2 years | 3 |  |
|  | 2 or more years but less than 5 years | 4 |  |
|  | 5 or more years | 5 |  |
|  | Never received dental care | 6 *If Never, go to O10* |  |
| What was the **main** **reason for your last visit** to the dentist? | Consultation / advice | 1 | O9 |
|  | Pain or trouble with teeth, gums or mouth | 2 |  |
|  | Treatment / Follow-up treatment | 3 |  |
|  | Routine check-up treatment | 4 |  |
|  | Other | 5 *If Other, go to O9other* |  |
|  | Other (please specify) | └─┴─┴─┴─┴─┘ | O9other |

| **Oral Health,** Continued | | | | | | | |
| --- | --- | --- | --- | --- | --- | --- | --- |
| **Question** | | **Response** | | | | **Code** | |
| How **often do you clean** your teeth? | | Never | | 1 If *Never, go to O14a* | | O10 | |
|  |  | Once a month | | 2 | |  |  |
|  |  | 2-3 times a month | | 3 | |  |  |
|  |  | Once a week | | 4 | |  |  |
|  |  | 2-6 times a week | | 5 | |  |  |
|  |  | Once a day | | 6 | |  |  |
|  |  | Twice or more a day | | 7 | |  |  |
| Do you use **toothpaste** to clean your teeth? | | Yes | | 1 | | O11 | |
|  |  | No | | 2 *If No, go to O13a* | |  |  |
| Do you use **toothpaste** containing **fluoride**? | | Yes | | 1 | | O12 | |
|  |  | No | | 2 | |  |  |
|  |  | Don't know | | 77 | |  |  |
| Do you use any of the following to **clean your teeth**?  *(RECORD FOR EACH)* | |  | |  | |  | |
| Toothbrush | | Yes | | 1 | | O13a | |
|  |  | No | | 2 | |  |  |
| Wooden toothpicks | | Yes | | 1 | | O13b | |
|  |  | No | | 2 | |  |  |
| Plastic toothpicks | | Yes | | 1 | | O13c | |
|  |  | No | | 2 | |  |  |
| Thread (dental floss) | | Yes | | 1 | | O13d | |
|  |  | No | | 2 | |  |  |
| Charcoal | | Yes | | 1 | | O13e | |
|  |  | No | | 2 | |  |  |
| Chewstick / miswak | | Yes | | 1 | | O13f | |
|  |  | No | | 2 | |  |  |
| Other | | Yes | | 1  *If Yes, go to O13other* | | O13g | |
|  |  | No | | 2 | |  |  |
| Other (please specify) | | └─┴─┴─┴─┴─┴─┴─┘ | | | | O13other | |
| Have you **experienced any of the following problems** during the past 12 months because of the **state of your teeth, gums or mouth**?  *(RECORD FOR EACH)* | |  | |  | |  | |
| Difficulty in chewing foods | | Yes | | 1 | | O14a | |
|  |  | No | | 2 | |  |  |
| Difficulty with speech/trouble pronouncing words | | Yes | | 1 | | O14b | |
|  |  | No | | 2 | |  |  |
| Mouth feels dry | | Yes | | 1 | | O14c | |
|  |  | No | | 2 | |  |  |
| Have a persistent wound and/or swelling in the mouth for more than three weeks | | Yes | | 1 | | O14d | |
|  |  | No | | 2 | |  |  |
| Have a red or red and white patch in the mouth | | Yes | | 1 | | O14e | |
|  |  | No | | 2 | |  |  |
| Felt tense because of problems with teeth or mouth | | Yes | | 1 | | O14f | |
|  |  | No | | 2 | |  |  |
| Embarrassed about appearance of teeth | | Yes | | 1 | | O14g | |
|  |  | No | | 2 | |  |  |
| Avoid smiling because of teeth | | Yes | | 1 | | O14h | |
|  |  | No | | 2 | |  |  |
| Sleep is often interrupted | | Yes | | 1 | | O14i | |
|  |  | No | | 2 | |  |  |
| Days not at work because of teeth or mouth | | Yes | | 1 | | O14j | |
|  |  | No | | 2 | |  |  |
| Difficulty doing usual activities | | Yes | | 1 | | O14k | |
|  |  | No | | 2 | |  |  |
| Less tolerant of spouse or people close to you | | Yes | | 1 | | O14l | |
|  |  | No | | 2 | |  |  |
| Reduced participation in social activities | | Yes | | 1 | | O14m | |
|  |  | No | | 2 | |  |  |

| **Step 2 Physical Measurements** |
| --- |

| **CORE: Blood Pressure** | | | |
| --- | --- | --- | --- |
| **Question** | **Response** | | **Code** |
| Interviewer ID |  | └─┴─┴─┘ | M1 |
| Device ID for blood pressure |  | └─┴─┘ | M2 |
| Cuff size used | Small | 1 | M3 |
|  | Medium | 2 |  |
|  | Large | 3 |  |
| Reading 1 | Systolic (mmHg) | └─┴─┴─┘ | M4a |
|  | Diastolic (mmHg) | └─┴─┴─┘ | M4b |
| Reading 2 | Systolic (mmHg) | └─┴─┴─┘ | M5a |
|  | Diastolic (mmHg) | └─┴─┴─┘ | M5b |
| Reading 3 | Systolic (mmHg) | └─┴─┴─┘ | M6a |
|  | Diastolic (mmHg) | └─┴─┴─┘ | M6b |
| During the past two weeks, have you been treated for elevated blood pressure with drugs (medication) prescribed by a doctor or other health worker? | Yes | 1 | M7 |
|  | No | 2 |  |
| **CORE: Height and Weight** | | | |
| **For women:** Are you pregnant? | Yes | 1 *If Yes, go to M 16* | M8 |
|  | No | 2 |  |
| Interviewer ID |  | └─┴─┴─┘ | M9 |
| Device IDs for height and weight | Height | └─┴─┘ | M10a |
|  | Weight | └─┴─┘ | M10b |
| Height | in Centimetres (cm) | └─┴─┴─┘. └─┘ | M11 |
| Weight  *If too large for scale 666.6* | in Kilograms (kg) | └─┴─┴─┘.└─┘ | M12 |
| **CORE: Waist** | | | |
| Device ID for waist |  | └─┴─┘ | M13 |
| Waist circumference | in Centimetres (cm) | └─┴─┴─┘.└─┘ | M14 |

| **EXPANDED: Hip Circumference and Heart Rate** | | | | | | | |
| --- | --- | --- | --- | --- | --- | --- | --- |
| Hip circumference | | in Centimetres (cm) | | └─┴─┴─┘.└─┘ | | M15 | |
| Heart Rate | | | | | |  | |
| Reading 1 | | Beats per minute | | └─┴─┴─┘ | | M16a | |
| Reading 2 | | Beats per minute | | └─┴─┴─┘ | | M16b | |
| Reading 3 | | Beats per minute | | └─┴─┴─┘ | | M16c | |

| **Step 3 Biochemical Measurements** |
| --- |

| **CORE: Blood Glucose** | | | |
| --- | --- | --- | --- |
| **Question** | **Response** | | **Code** |
| During the past 12 hours have you had anything to eat or drink, other than water? | Yes | 1 | B1 |
|  | No | 2 |  |
| Technician ID |  | └─┴─┴─┘ | B2 |
| Device ID |  | └─┴─┘ | B3 |
| Time of day blood specimen taken (24-hour clock) | Hours: minutes | └─┴─┘: └─┴─┘  hrs mins | B4 |
| Fasting blood glucose  *[CHOOSE ACCORDINGLY: MMOL/L OR MG/DL]* | mmol/l | └─┴─┘. └─┴─┘ | B5 |
|  | mg/dl | └─┴─┴─┘.└─┘ |  |
| Today, have you taken insulin or other drugs (medication) that have been prescribed by a doctor or other health worker for elevated blood glucose? | Yes | 1 | B6 |
|  | No | 2 |  |
| **CORE: Blood Lipids** | | | |
| Device ID |  | └─┴─┘ | B7 |
| Total cholesterol  *[CHOOSE ACCORDINGLY: MMOL/L OR MG/DL]* | mmol/l | └─┴─┘. └─┴─┘ | B8 |
|  | mg/dl | └─┴─┴─┘.└─┘ |  |
| During the past two weeks, have you been treated for elevated cholesterol with drugs (medication) prescribed by a doctor or other health worker? | Yes | 1 | B9 |
|  | No | 2 |  |
| **CORE: Urinary sodium and creatinine** | | | |
| Had you been fasting prior to the urine collection? | Yes | 1 | B10 |
|  | No | 2 |  |
| Technician ID |  | └─┴─┴─┘ | B11 |
| Device ID |  | └─┴─┘ | B12 |
| Time of day urine sample taken (24-hour clock) | Hours: minutes | └─┴─┘: └─┴─┘  hrs mins | B13 |
| Urinary sodium | mmol/l | └─┴─┴─┘.└─┘ | B14 |
| Urinary creatinine | mmol/l | └─┴─┘. └─┴─┘ | B15 |

| **EXPANDED: Triglycerides and HDL Cholesterol** | | | |
| --- | --- | --- | --- |
| **Question** | **Response** | | **Code** |
| HDL Cholesterol  *[CHOOSE ACCORDINGLY: MMOL/L OR MG/DL]* | mmol/l | └─┘. └─┴─┘ | B16 |
|  | mg/dl | └─┴─┴─┘.└─┘ |  |
|  |  |  |  |
